# Molecular Imaging Probes Based on Matrix Metalloproteinase Inhibitors (MMPIs)

**DOI:** 10.3390/molecules24162982

**Published:** 2019-08-16

**Authors:** Loganathan Rangasamy, Bruno Di Geronimo, Irene Ortín, Claire Coderch, José María Zapico, Ana Ramos, Beatriz de Pascual-Teresa

**Affiliations:** Departamento de Química y Bioquímica, Facultad de Farmacia, Universidad San Pablo-CEU, CEU Universities, Urbanización Montepríncipe, Alcorcón, 28925 Madrid, Spain

**Keywords:** Matrix metalloproteinases (MMPs), positron emission tomography (PET), single-photon emission computed tomography (SPECT) and optical imaging (OI)

## Abstract

Matrix metalloproteinases (MMPs) are a family of zinc- and calcium-dependent endopeptidases which are secreted or anchored in the cell membrane and are capable of degrading the multiple components of the extracellular matrix (ECM). MMPs are frequently overexpressed or highly activated in numerous human diseases. Owing to the important role of MMPs in human diseases, many MMP inhibitors (MMPIs) have been developed as novel therapeutics, and some of them have entered clinical trials. However, so far, only one MMPI (doxycycline) has been approved by the FDA. Therefore, the evaluation of the activity of a specific subset of MMPs in human diseases using clinically relevant imaging techniques would be a powerful tool for the early diagnosis and assessment of the efficacy of therapy. In recent years, numerous MMPIs labeled imaging agents have emerged. This article begins by providing an overview of the MMP subfamily and its structure and function. The latest advances in the design of subtype selective MMPIs and their biological evaluation are then summarized. Subsequently, the potential use of MMPI-labeled diagnostic agents in clinical imaging techniques are discussed, including positron emission tomography (PET), single-photon emission computed tomography (SPECT) and optical imaging (OI). Finally, this article concludes with future perspectives and clinical utility.

## 1. Introduction

Molecular imaging is a rapidly emerging multidisciplinary field that can visualize physiological or pathological processes inside the body at the cellular or molecular level. Molecular imaging plays an important role in noninvasive earlier diagnosis, the accurate detection of diseases or dysfunctions, treatment follow-ups, personalized treatments, and it is also useful in drug development and discovery processes [1]. There are several modalities available for clinical imaging purposes, including positron emission tomography (PET), single photon emission computed tomography (SPECT), magnetic resonance imaging (MRI), X-ray computed tomography imaging (CT), optical imaging (OI) by either bioluminescence or fluorescence, and ultrasound (US). Each imaging modality has advantages and disadvantages in aspects, such as spatial resolution, penetration depth and sensitivity which are collected in Table 1 [2]. In recent years, many efforts have been made to improve the imaging instruments and advanced image reconstruction techniques to obtain high resolution images that disclose even tiny lesions and gather accurate quantification of pathological processes. Both academia and the pharmaceutical industry are making a great effort to develop novel molecular imaging probes. The ideal molecular imaging probe must bind to its target with enough affinity, have optimal circulation time inside the body, rapidly clear from the tissues and present high metabolic stability. There are numerous molecular imaging agents available both for preclinical and clinical applications, based on small molecules, peptides, affibodies, aptamers, antibodies and nanoparticles [2]. Among these agents, small molecule-based probes remain promising candidates for clinical applications, because they have favorable pharmacokinetic and pharmacodynamic profiles, generally have short circulation times, and are rapidly cleared from the body, thereby reducing the possibility of off-target toxicity. The design and development of molecular imaging probes are unlikely be successful unless the molecular target is well defined, and its biological role is well characterized. According to this requirement, matrix metalloproteinases (MMPs) are highly validated molecular targets for the development of both therapeutics and diagnostic agents [3,4,5,6]. MMPs are frequently found to be overexpressed or highly activated in numerous human diseases, including cancer, atherosclerosis, stroke, arthritis, cardiovascular diseases, periodontal disease, respiratory tract disorders, glomerulonephritis, abdominal aortic aneurysm expansion, inflammatory bowel disease, neurodegeneration, chronic obstructive pulmonary disease, multiple sclerosis and liver fibrosis [7,8,9,10,11]. MMPs represent valuable targets for the development of new therapeutic and diagnostic tools. Owing to the important role of MMPs in many human diseases, several MMP inhibitors (MMPIs) have been developed as novel therapeutics and a few of them namely, Marimastat, BB-1101, Rebimastat, Prinomastat, RO-32-3555, Ilomastat, CGS-27023A, Batimastat, Tanomastat, among others have entered in advanced stages of clinical trials. However, many MMPIs have failed in the last phases of clinical trials mainly due to musculoskeletal toxicity, joint pain and swelling, as well as bone marrow suppression and venous thromboembolism. These side-effects could be due to the high concentrations of inhibitors required for the therapy [3,4,5,6]. However, they may be useful for the development of imaging probes because even low concentrations of tracers would be enough to image the activated MMPs in preclinical and clinical use (e.g., in a preclinical model).

Hence, radio-/fluorophore- labeled small molecule MMPIs could be useful for the detection of activated MMPs by means of molecular imaging techniques, such as SPECT, PET and OI. In recent years, numerous labelled MMPIs imaging agents have emerged. This review summarizes the MMP-based imaging probes for diseases listed above. The labelled MMPIs imaging agents based on the specific disease application and on their structure have been organized. An intensive broad discussion has been made on the synthetic methods including the yield, purity, overall synthesis time and specific activity of the probes, in vitro MMP isoform selective binding affinity, in vitro imaging, in vivo biodistribution, metabolic stability assessment, and in vivo preclinical to human clinical imaging applications. This review specifically covers the development of small-molecule PET, SPECT and OI probes but does not include MRI imaging probes, which have been reviewed elsewhere [3,4,5,6]. The experimental details on the radiosynthesis of MMPIs in Table 2 have been summarized. This review does not cover the MMP activatable probes. This work widens and updates previous reviews [6] reported in the literature which focused only on one disease [4,5] or one enzyme [3].

### 1.1. Classifications and Structures of Matrix Metalloproteinases

**Figure 1:**
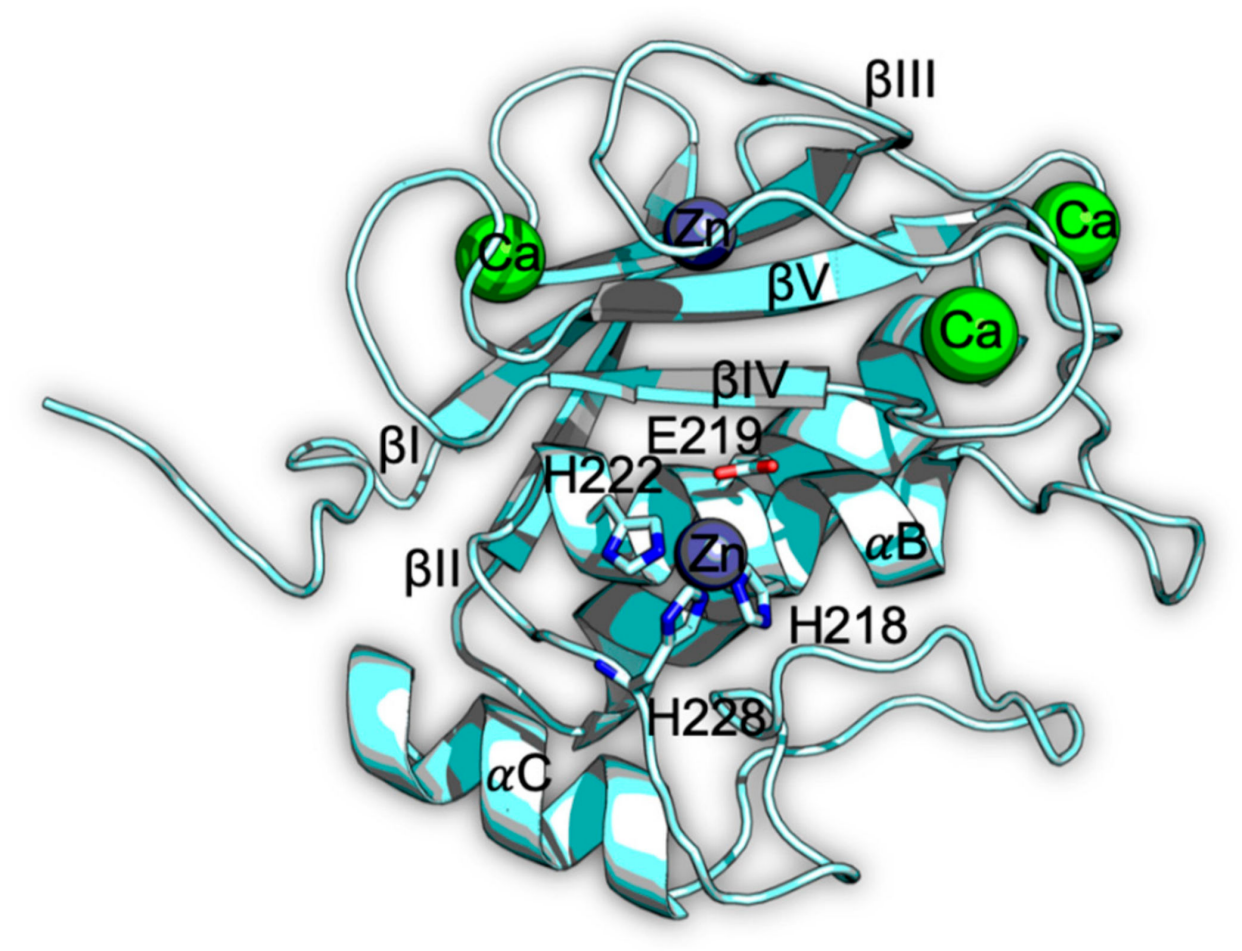
MMP Catalytic subunit. A cartoon representation of the MMP-1 (PDB code 1CGF) showing the different α-helixes and β-sheets with the corresponding coordinated metal ions. The sticks highlight the active site with the three histidines and the catalytic glutamic acid.

During the last 40 years, MMPs have become an attractive pharmacological target due to their relationship with several diseases, especially in cancer invasion and metastasis [12]. The main role of these zinc-dependent endopeptidases lies not only in their ability to remodel the extracellular matrix (ECM) by degrading the connective tissue, but also in their involvement in contact cell regulation [13]. The normal function of these enzymes is related to tissue homeostasis by maintaining the complex ECM and preserving its biochemical and mechanical properties. Therefore, a dysregulation in MMP activity could lead to both the development and cancer progression, as well as other diseases such as atherosclerosis, osteoarthritis, pulmonary and cardiovascular diseases [14]. The entire MMP genome includes 24 distinct genes encoding for more than 26 members. These have been classified based on the substrate type (collagenases, gelatinases, stromelysins, matrilysins, membrane-type MMPs and others) or by their structure [15]. Structurally distinct MMPs such as MMP-2, -7, -8, -9, -12 and -13 have been targeted with MMPIs labelled as diagnostic agents [6]. MMPs are synthesized and secreted as preproenzymes (zymogens) apart from MMP-8 and MMP-9 that are present in neutrophils and located inside granulomas. The activation process typically occurs extracellularly by proteolytic cleavage by serine proteinases or even by other MMPs. Commonly, MMPs are multi-domain proteins, which contain a signal sequence that targets the peptide for secretion; a propeptide responsible for the inactivation; a catalytic domain; a linker domain (also called a hinge region); and a hemopexin-like domain [16]. The propeptide is made up by the PRCGXPD motif that can inhibit the enzyme by a Cys-Zn^2+^ chelation that occludes the active site. Apart from the auto inhibition by tissue inhibitors of matrix metalloproteinases (TIMPs) and the general plasma proteinase inhibitor, α2-macroglobulin can also regulate MMPs activity [17]. The MMP catalytic domain consists of a globular domain formed by approximately 170 amino acids where five central β-sheets are wrapped around four α-helixes. These metalloenzymes contain two zinc ions: One is part of the catalytic site and is coordinated by three histidines contained in the well conserved zinc-binding motif (HEXXHXXGXXH); the second exerts structural functions and its absence is linked to an autoinhibition allosteric process [18]. Furthermore, at least 2 or 3 calcium ions having structural functions are present depending on the 3D-crystal structure. Furthermore, the general structure presents five β-sheets (βI–βV) and three α-helixes (αA, αB and αC) within the N-terminal subdomain. The catalytic site is located in the αB (Figure 1). By linking αB and αC, a notable Ω-loop is found, presenting a variety of lengths and amino acid compositions depending on the type of MMP. The Ω-loop has become one of the paradigms in MMP drug discovery, as it is the least conserved fragment within the catalytic domain that can offer different ligand selectivity [19]. There are several hydrophobic pockets described in the catalytic domain of MMPs which are designated by a primed or unprimed S subsite. As described by Babine and Bender [20,21], the subsites on the left side of the Zn^2+^ ion are designated without a prime S (S1, S2, S3..), while those present on the right side are primed (S1´, S2´, S3´…) and the numbers refer to the residue numbers present on the substrate that occupy the pockets being the first ones cleaved by the MMP and 2 and 3 the adjacent.

The S1’ pocket is a highly hydrophobic cavity formed by the Ω-loop. Although this subsite is present in all MMPs, its volume and plasticity varies depending on the nature of the Ω-loop, giving rise to a MMP classification according to the size of the S1’ pocket: Small (MMP-1, MMP-7, MMP-20, MMP-22), medium (MMP-2, MMP-8, MMP-9, MMP-12, MMP-14, MMP-16), and large (MMP-3, MMP-10, MMP-13) [22]. The S1’ pocket is the major hydrophobic cavity near the active site and has attracted major attention in the design of selective MMP inhibitors because of its variation in size and depth [23]. Furthermore, another side pocket has been described, the S1’*, which extends beyond the S1’ pocket, and its accessibility depends also on the position and conformation of the Ω-loop. The presence of this extra pocket has been only mentioned in some MMPs, such as MMP-8 and MMP-13 [24,25]. In addition to the S1’ and S1’* pockets, the S1, S2, S3 and S2′, S3’ subsites can also offer some degree of selectivity [21]. 

### 1.2. MMPs as Biomarkers in Cancer, Atherosclerosis, Osteoarthritis, Pulmonary and Cardiovascular Diseases

Although the knowledge that MMP overexpression has an influence in the risks and prognosis of several types of cancer and other diseases is not new, the application of MMPs as biomarkers is relatively recent. An ideal biomarker presents the capacity to be measured and evaluated as an indicator of the biological process of the target. Therefore, a biomarker should be accurate, non-invasive, sensitive, non-expensive and easy to perform [26]. The recently developed biomarkers are deficient in specificity and sensibility when they are applied in a single combination, so nowadays biomarker combination is one of the strategies to overcome these weaknesses [27]. 

Currently, MMPs are being explored as biomarkers in several fields such as diagnosis, monitoring and treatment efficacy in different diseases. In this line, their application in cancer, atherosclerosis, osteoarthritis, and pulmonary and cardiovascular diseases has been remarkable. This novel approach comes mainly because the overexpression of MMPs in these diseases is specific and elevated. Moreover, these enzymes are available in the extracellular media, which means the biomarker molecule does not need to cross the cell membrane. An extensive review made by Huang et al. [28] summarized how MMP-9 can be explored as a potential biomarker, and also how this MMP is overexpressed in several types of tumors such as colorectal carcinoma, breast, pancreatic, ovarian, cervical, osteosarcoma non-small cell lung cancer (NSCLC) and giant cell tumor of bone (GCTB). This makes MMP-9 a preferential candidate for the early detection of these types of tumors. The elevated MMP-9 expression in those tumors compared with healthy controls can be detected due to the recent development of experimental analytical techniques such as proteomic analysis, immunohistochemical studies, immunochemistry assays and tandem mass spectrometry [29,30,31]. The augmented levels of MMP-9 can be detected in plasma or blood samples, showing triple protein levels compared to healthy patients. The study carried out by Zajkowska et al. [29] showed that patients with cervical cancer in stages III and IV present MMP-9 plasma levels of 3344.44 ng/mL and 325.80 ng/mL, respectively, with almost three times more compared to healthy patients which present with normal levels of 166.00 ng/mL. De Chiara et al. [32] measured the MMP-9 levels in blood samples and found them to be triple (302.92 ng/mL) for NSCLC patients than in healthy controls (136.14 ng/mL). By tissue sampling, the overexpression of MMP-9 has also been detected by immunohistochemical assays using micro-RNA and labelled IgG in different types of cancer such as colorectal carcinoma, and breast cancer. Moreover, this overexpression has been linked to metastatic processes [30,31]. In the case of GCTB, the resection after surgery was the only available option to get the samples in order to measure the MMP-9 mRNA expression in peripheral tissue, confirming the overexpression in this type of cancer. These published diagnostic screenings in MMP-9 present as the main drawback the lack of tumor localization for blood samples and the inconvenience of the biopsy sampling. This gives an idea of the potential of MMP-9 as a biomarker. 

Further, MMP-2, -7, -11, and -12, have been reported to be involved in cell growth and metastasis processes. In this line, MMP-12 overexpression has been linked to the grade and stage of esophageal squamous cell carcinoma in tissue sampling [33]. This protease has also been connected with the exposure to tobacco smoke and with gefitinib-resistant NSCLC that leads to a poor prognosis [34,35]. Related to its implication in the effects of the exposure to cigarette smoke, MMP-12 is a key enzyme in diseases such as chronic obstructive pulmonary disease (COPD) and has been proposed as an early biomarker due to its elevated levels in tissue sampling [36,37]. 

MMP-11 has promoted the occurrence and development of malignant tumors such as breast [38], gastric [39], colorectal (CRC) [40], and lung cancer [41]. Similarly, MMP-7 overexpression has been correlated with the incidence of gastric cancer. The growth/differentiation factor 15 overexpression was also linked with this type of cancer, showing almost more than ten times the protein level compared with healthy patients [42]. MMP-7 has been described to play critical roles in the development and progression of other malignant tumors including CRC [43,44] and triple-negative breast cancer [45], amongst others. In conclusion, MMP-7 has been designated as a tumor suppressor. MMP-2 has been involved in the migration and invasion of ovarian, colorectal, bladder and breast cancer, where the increased activity and expression of these enzymes have been described [46,47,48,49]. Additionally, a remarkable synergic effect between Cullin1 and MMP-2 in CRC prompt diagnosis has been proposed by Deng et al. An extensive study with 470 patients showed that protein levels were significantly upregulated in CRC tissues compared to the healthy ones [50]. However, the overexpression of MMP-2 has been linked with poor prognosis in patients with epithelial ovarian and lung cancers [51,52]. 

On the other hand, MMPs have also been involved in the development and progression of atherosclerosis, which is correlated with cardiovascular diseases, such as a stroke and ischemic heart diseases, two of the top worldwide causes of death. The activity of these proteases promotes the loss of collagen, elastin, and other ECM proteins, inducing the necrotic core of atherosclerotic plaque, which finally could end in myocardial infarction or a stroke [10]. It has been shown that increased levels of MMP-9 are present in specific risk groups, like obese people that present a profile for early atherosclerosis development [53]. Furthermore, Rohde et al. demonstrated that MMP-9 serum levels were increased in patients with definite carotid atherosclerotic disease compared with normal patients; and also, that only MMP-9 levels were increased in contrast with other proposed biomarkers such as MMP-1 and MMP-3 [54]. A study carried out by Nilsson et al. [55] using a cohort of 1500 patients, and measuring the plasma levels of MMP-1, -3, -7, -10, and -12, demonstrated that MMP-7 and MMP-12 were elevated in type 2 diabetes, which is related to atherosclerosis and coronary events. Another meta-study carried out by Zhong et al. [56] in which the serum levels of MMP-1 and TIMP were evaluated in patients with atrial fibrillation (AF), demonstrated that elevated mRNA levels of MMP-1 and decreased circulating levels of TIMP2 were significantly associated with AF risk. 

Osteoarthritis (OA) is also one of the diseases linked to MMP overexpression due to their implication in the breakdown of articular cartilage in synovial joint tissues. Many publications have linked OA to the overexpression of MMPs in the affected tissue, especially MMP-1, -2, -9 and -13 [57,58]. MMP-1 is proposed as an early OA biomarker due to its increased concentrations in the synovial fluid in the early stage of OA compared to decreased levels when OA continues progressing [59]. Remarkably, MMP-13 has raised interest in clinical studies, as it is overexpressed in the cartilage of patients with OA and is identified as a target protein in the signaling pathways implicated in the regulation of cartilage breakdown [60,61]. MMP-3 has also been proposed as an OA biomarker together with other pro-inflammatory enzymes due to its markedly up-regulation in this disease [62]. 

## 2. MMPIs Labelled Imaging Agents for Cancer

### 2.1. CGS 27023 and CGS 29566 Based MMPIs Labelled Imaging Probes

Several MMPIs such as Batimastat, Marimastat, Tanomastat, Prinomastat (AG-3340), Rebimastat, CGS 25966A, and CGS 27023A have entered human trials. Among these, CGS 27023A presents both good pharmacokinetics and IC50 in the nanomolar range for several MMP isoforms.

**Scheme 1:**
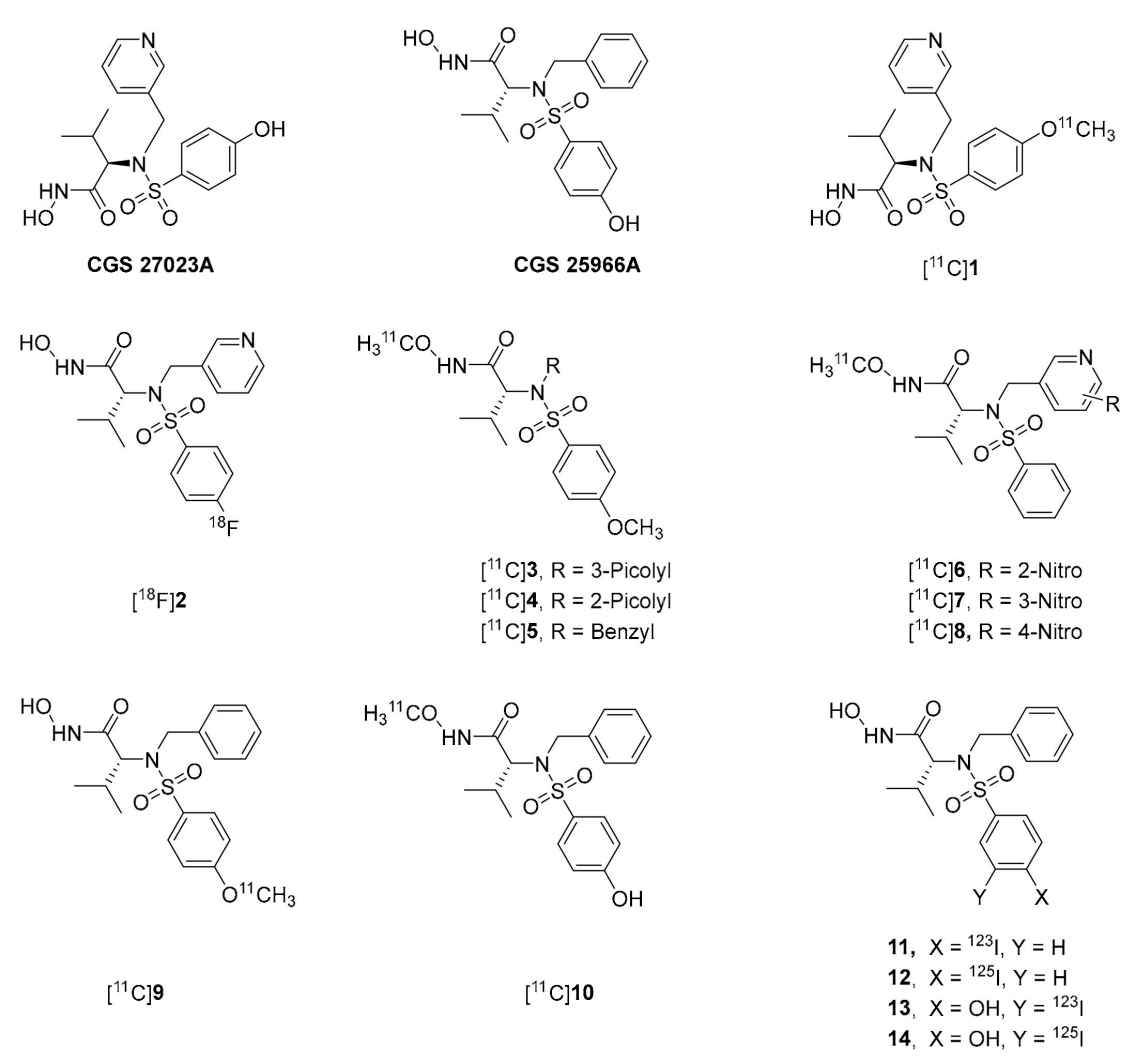
Chemical structure for compounds CGS 25966A, CGS 27023A and **1**–**14**.

**Scheme 2:**
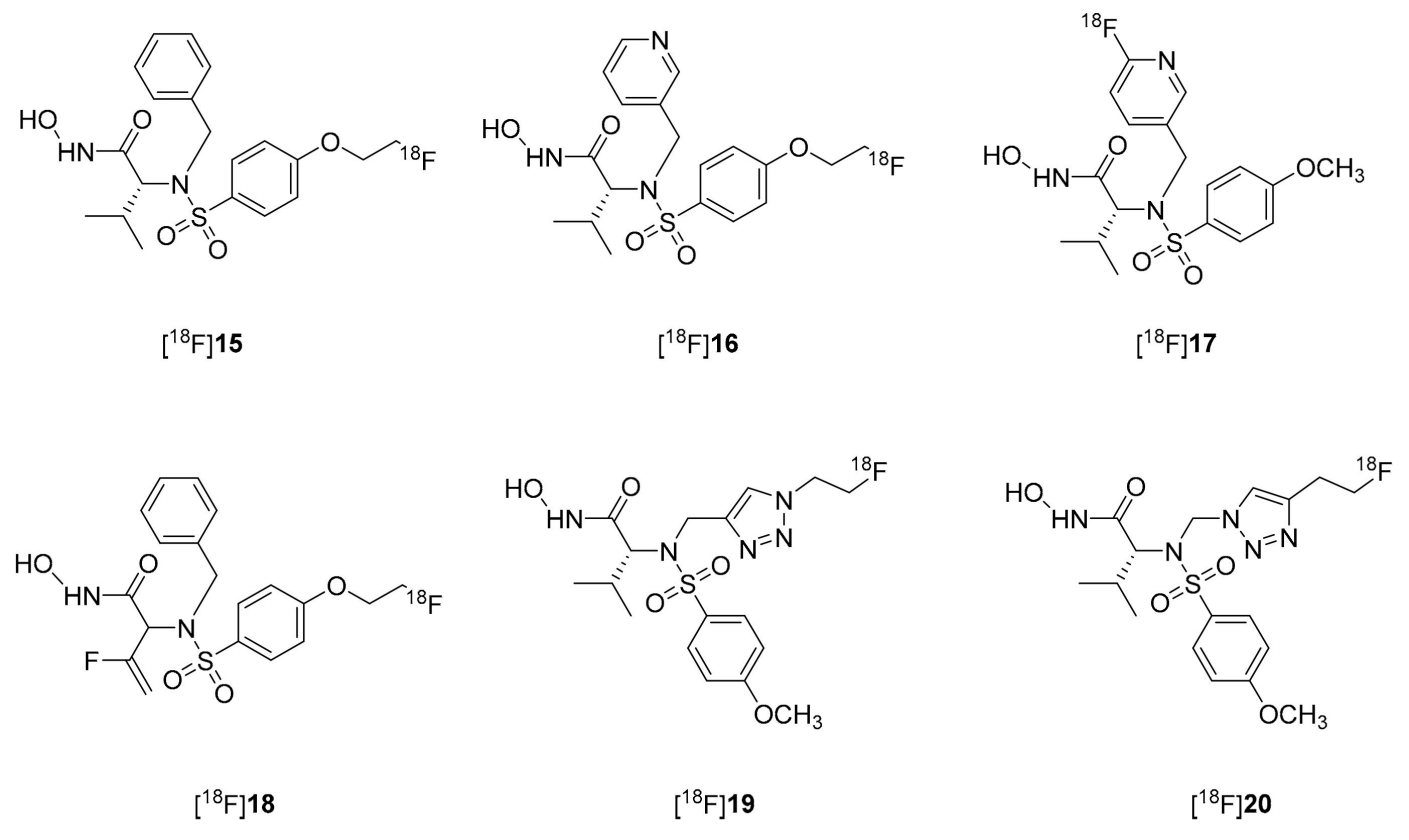
Chemical structure for compounds **15**–**20**.

Inspired by the MMP inhibitory activity of CGS 27023A, Zheng et al. [63] designed a number of ^11^C or ^18^F labeled analogs such as [^11^C]**1**, [^18^F]**2** and [^11^C]**3**. These are the first MMPI radiotracers described in the literature [63]. Later on, the same research group reported analogs [^11^C]**4**–[^11^C]**8**, as radiolabeled MMPIs [64]. However, no reports of in vivo applications of [^11^C]**4**–[^11^C]**8** have been found. Further, based on CGS 25966A, Zheng et al. [65] reported simple synthetic methods for [^11^C]**9** and its isomer [^11^C]**10**. The novel no-carrier-added radioiodinated MMPIs [^123^I]**11**, [^125^I]**12**, [^123^I]**13** and [^125^I]**14** were prepared by radioiodination of their precursors with [^123^I]NaI or [^125^I]NaI (Scheme 1) [66]. Preliminary in vitro data showed affinities towards MMP-2 and MMP-9 in the lower nanomolar range comparable to those of the parent compound CGS 27023A. In vivo biodistribution using [^125^I]**14** in CL57 B/6 mice for 65 min p.i. showed rapid blood and plasma clearance and low retention in normal tissues. This is indicative of the suitability of these radiotracers for imaging MMP activity in vivo. However, in vivo studies using animal models that represent diseases with known MMP overexpression (e.g., tumor: Lewis lung carcinoma bearing mice; atherosclerotic plaques: apolipoprotein E-deficient mice) are needed to support their observation. Wagner et al. described a series of [^18^F]-labeled compounds ([^18^F]**15**–**20**) (Scheme 2) [67]. Compound [^18^F]**16** presented 43-fold increased potency compared to the parent compound CGS 27023A. Neither of these two compounds presented a tissue-specific accumulation in wild type (WT) mice, which can be considered an advantage in the study of MMP dysregulation. Therefore, compounds [^18^F]**15** and [^18^F]**16** could be useful for the in vivo imaging of activated MMPs in inflammation, cancer or atherosclerosis.

The same group reported a fully automated robust and reproducible radiosynthesis for [^18^F]**15** with reasonable yields, whereas compound [^18^F]**17** was synthesized in a one-step radiosynthesis with moderate radiochemical yield. Despite the latter presenting a high lipophilicity (experimental log D = 1.85), the initial PET in vivo studies in WT mice (C57/BL6) showed neither unspecific accumulation of [^18^F]**17** in the heart or carotid regions, nor accumulation of radioactivity in the bones due to tracer decomposition. Compound [^18^F]**18** was obtained in its two enantiomerically pure (*R*)- and (*S*)-isomers, and both demonstrated to be potent fluorinated inhibitors of MMP-2 and MMP-9 [68]. Remarkably, the (*S*)-enantiomer was even more active than the (*R*)-isomer in contrast to CGS 25966. Unfortunately, (*S*)-[^18^F]**18** is metabolically unstable in WT mice, which hinders further clinical development of this tracer. Triazole-containing unlabeled precursors of compounds [^18^F]**19** and [^18^F]**20** [69], possess increased hydrophilicity compared to CGS 25966A (clog D values ranging from 0.60 to 2.25) and nanomolar inhibition potencies for MMP-2, MMP-8, MMP-9, and MMP-13 (see Table 2) [70].

Compound [^18^F]**19** showed an excellent serum stability in vitro, up to 120 min, and a rapid clearance through the hepatic and renal clearance routes without presenting unspecific binding to non-excretion organs. Although these results indicate that [^18^F]**19** is a promising MMP-targeted radiotracer, there is a lack of murine disease models characterized by the up-regulated levels of the activated MMPs to carry out PET/CT preclinical studies. Compound [^18^F]**20** exhibited excellent serum stability and rapid renal clearance. Despite no defluorination or nonspecific accumulation of [^18^F]**20** in non-excretory organs were observed in vivo, the biodistribution and metabolism studies in ICR (CD1) WT mice showed mainly polar metabolites of [^18^F]**20** [70].

### 2.2. Fluorescent Probes for Cancer

**Scheme 3:**
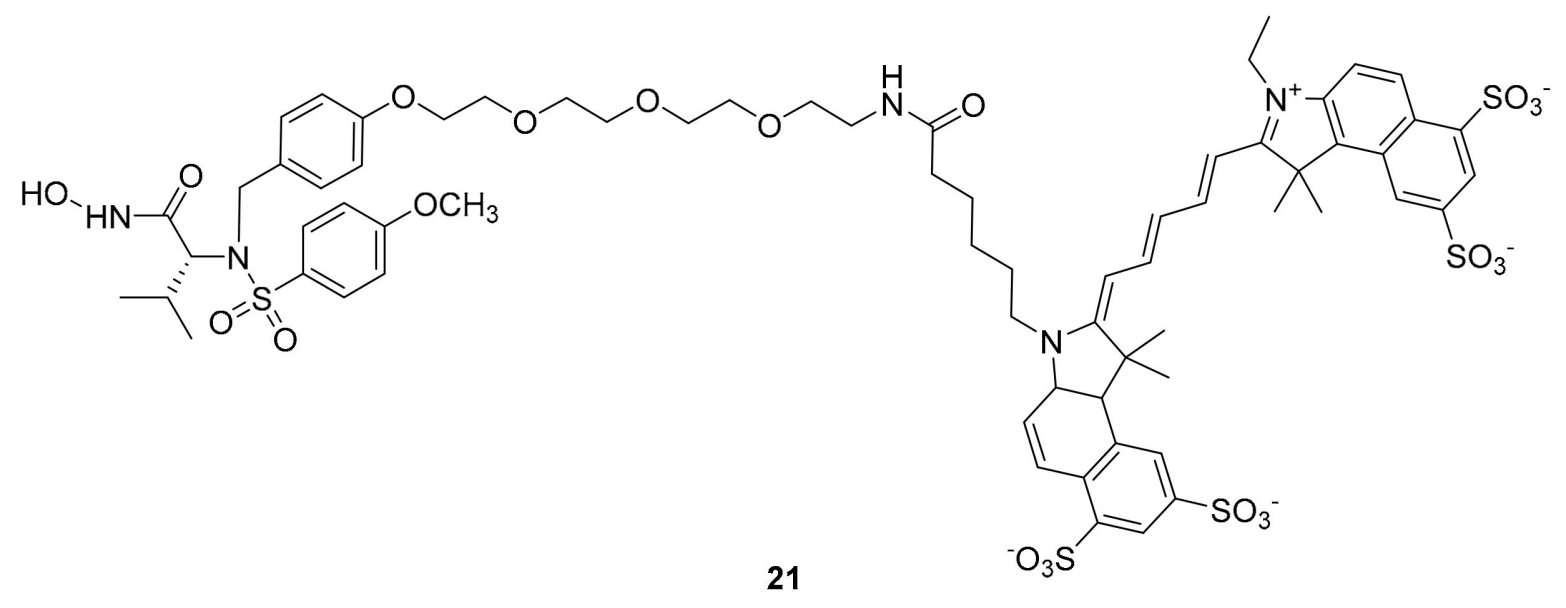
Chemical structure for compound **21**.

Faust et al. developed a hydroxamate based Cyanine5.5 labelled fluorescence imaging probe for MMPs [71]. Starting from CGS 27023A and CGS 25966A, they synthesized compound **21** after a 11 steps synthetic route. Before conjugating Cy5.5, the parent compound showed complete inhibition of MMP-2/-9 activities in vitro, both for purified MMP-2/-9 (active and pro-form) and MMP-2/-9 containing cell culture supernatants. However, as expected, the fluorescent probe **21** lost the nanomolar potency of its unlabeled precursor. In situ zymography studies were conducted cryostat sections of A-673 (rhabdomyosarcoma), HT1080 (fibrosarcoma), and BT-20 (breast cancer) xenograft (CD-1 nude mice), showing high, weak and low MMP-2 and -9 activities, respectively. The MMP positive tumor cryostat sections specifically showed a higher signal with compound **21** while MMP negative tumors were not targeted. Thus, compound **21** (Scheme 3) may be used for a sensitive imaging of MMP activity in various tumours. 

Waschkau et al. [72] studied the in vivo applications of **21** using four different tumor xenografts: A-673, HT-1080, MDA-MB 231, and BT-20 tumors. They all showed different levels of MMP-2 and -9 expression, as indicated by the reverse transcription polymerase chain reaction (RT-PCR) immunohistochemical analysis. The in vivo dynamic pharmacokinetic studies demonstrated that **21** was quickly cleared from the circulation. The ex-vivo biodistribution of **21** at 6 h p.i. showed a significant uptake in the tumor, kidney, lung and liver. After 24 h, the fluorescence intensity in most of the tissues was reduced by approximately 50%, while at 72 h, **21** was completely eliminated from all organs. The time-course near infrared fluorescence (NIRF) image in all the above four xenografts showed that the maximum tumor-to-background ratio values were observed between 45 min and 6 h. Then, the tumor-to-background ratio values were progressively reduced at 24 h and were almost negligible at 72 h in A-673 and HT-1080 tumors, proving its ability to visualize MMP specific activity in vivo. 

Interestingly, they compared **21** with commercially available macromolecule-based MMP-activatable probes, MMPSense^TM^ 680 and MMPSense^TM^ 750 FAST. The activatable probes require a long circulation time (24 h or longer) which is potentially incompatible with human applications. However, **21** gave high NIRF intensities for MMP-positive tumors 30 min after injection and was eliminated from all the organs at 72 h, which makes it applicable to in vivo experiments. Further, they revealed that the dual probe approach by injecting MMPSense^TM^ 750 FAST and **21** enabled the imaging of MMP-positive A-673 tumors at different time windows. For instance, **21** signals in A-673 tumors were clearly detectable at 30 min and retained up to 6 h, but MMPSense^TM^ 750 FAST signal was detectable only at 6 h. Therefore, compound **21** could be useful for non-invasively image tumoral MMP-2/-9 expression patterns. This non-peptidic low-molecular-weight fluorescent probe may be clinically translated for endoscopic or surgical procedures.

### 2.3. MMP Inhibitor Imaging Probes with Carboxylic Acid as Zing Binding Group (ZBG)

**Scheme 4:**
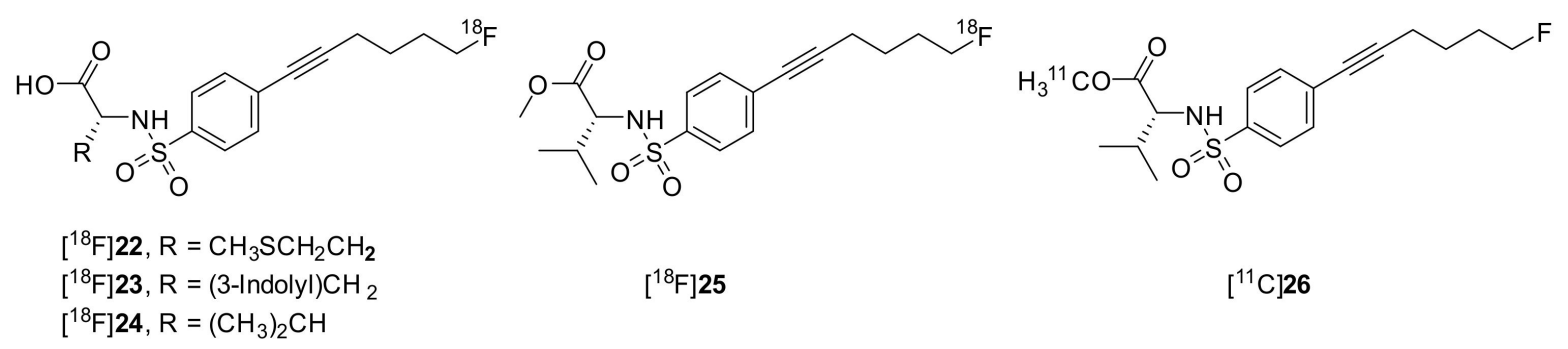
Chemical structure for compounds **22**–**26**.

Furumoto et al. [73] designed and developed ^18^F-labeled carboxylic acid-based MMPIs [^18^F]**22**–**24** for PET cancer imaging. The precursors were synthesized in four steps starting from the D-isomer of the corresponding amino acid. Although compound [^18^F]**22** was chemically unstable and thus unsuitable for biological applications, compounds [^18^F]**23** and [^18^F]**24** (Scheme 4) were isolated in moderate radiochemical yields, and their biological evaluation was reported by the same authors [74].

A biodistribution study in the Ehrlich tumor-bearing mice showed that the accumulation of [^18^F]**24** in tumor was higher than in other organs, except for the liver, small intestine, and bone. When [^18^F]**25**, a methyl ester of [^18^F]**24**, was used as a prodrug, the uptake in liver at 30 min p.i. decreased by half and the tumor uptake increased by 2.4 times, compared with [^18^F]**24**. A radio-thin-layer chromatographic analysis of [^18^F]**25** demonstrated that it can be easily converted to the parent drug in vivo and thus accumulated in the tumor tissue. Therefore, [^18^F]**25** could be considered a prodrug of [^18^F]**24**. Whole body autoradiography using [^18^F]**25** showed tumor-specific accumulation of radioactivity, with higher accumulation in the bone and intestine. However, the increase of the uptake in the bone was time-dependent, suggesting the accumulation of free ^18^F^−^ anion, released from the terminal alkyl chain of [^18^F]**25**. These results suggest that the prodrug strategy could be useful, however the release of free [^18^F^−^] should be avoided. 

**Figure 2:**
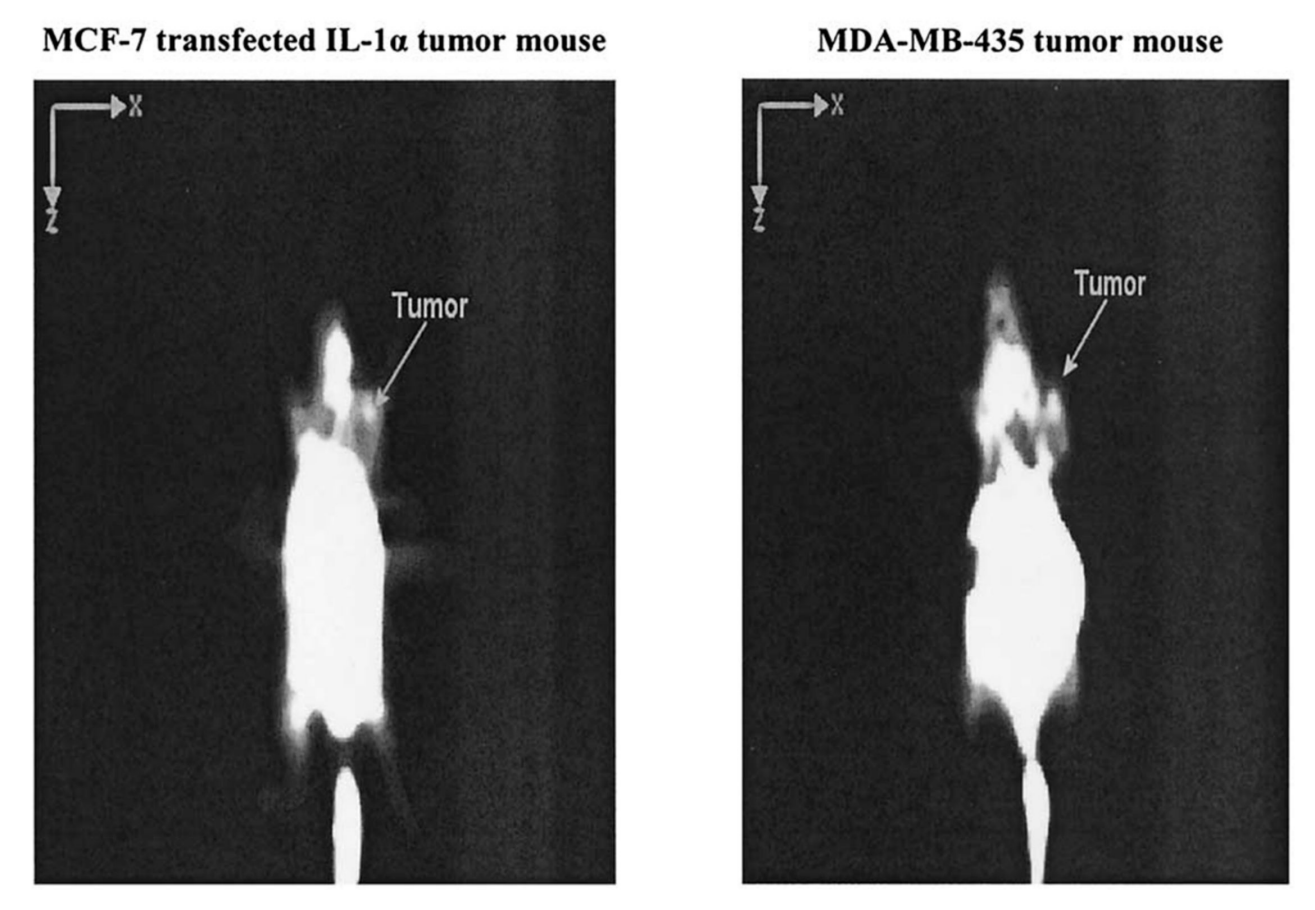
Micro-PET images of [^11^C]**26** in a MCF-7 transfected with IL-1α implanted athymic mouse, and a MDA-MB-435 implanted mouse, after an initial 30 min uptake period. Reprinted with permission from [75] Copyright © 2003 Elsevier Inc. All rights reserved.

Structurally related compound [^11^C]**26** has been studied as a potential PET cancer tracer [75]. The biodistribution and micro-PET studies in breast cancer animal models, such as MCF-7 transfected with IL-1α implanted athymic mice, and MDA-MB-435 implanted athymic mice confirmed that the localization of [^11^C]**26** in the tumor was mediated by non-specific processes (Figure 2). 

### 2.4. Biphenyl Sulfonamide Based MMPIs Labelled Imaging Probes

**Scheme 5:**
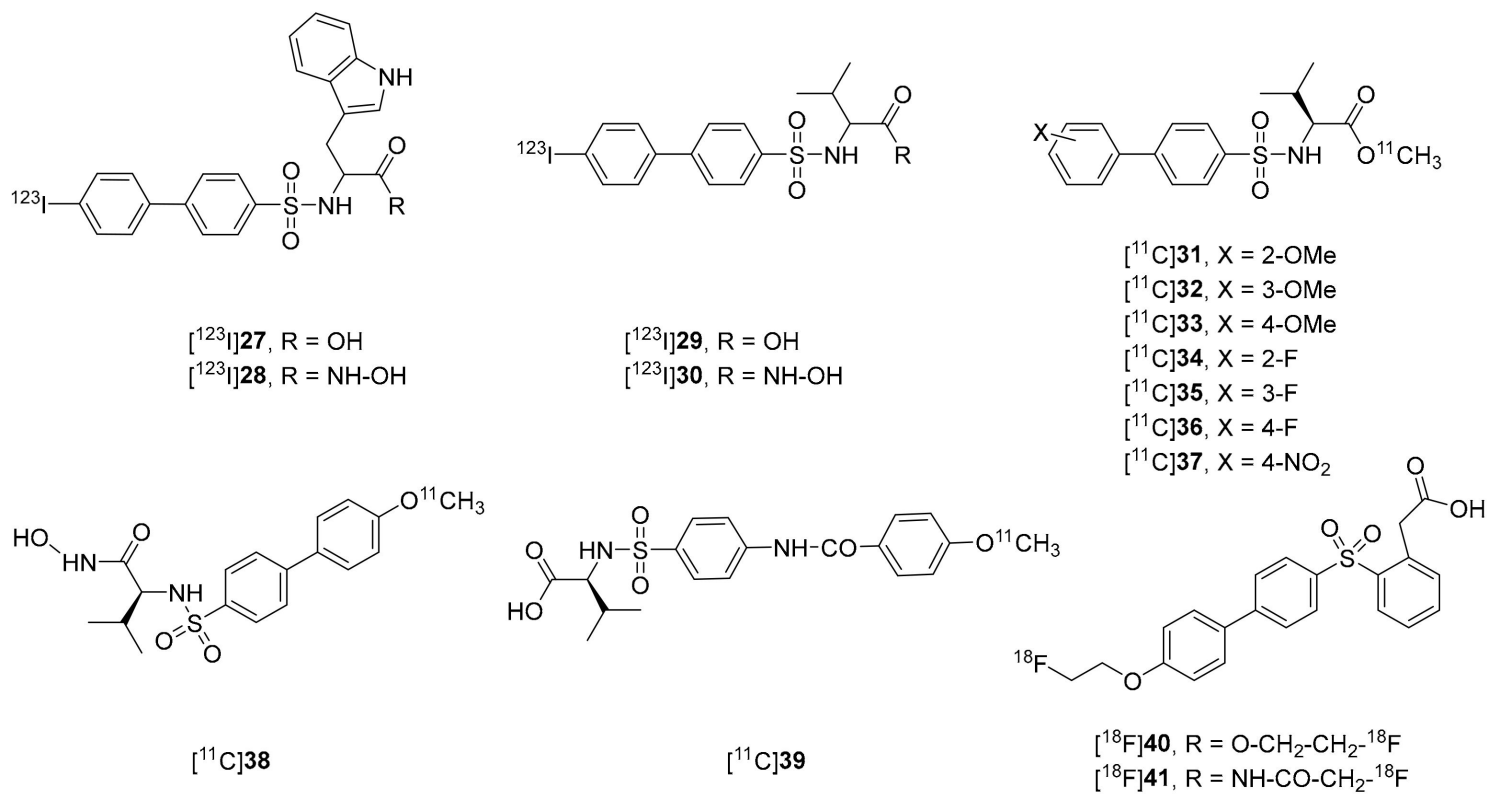
Chemical structure for compounds **27**–**41**.

Oltenfreiter et al. developed a series of radioiodinated carboxylic and hydroxamic acids [^123^I]**27**–**30** (Scheme 5) [76,77,78]. 

The in vitro zymography and enzyme assays showed IC_50_ in the nanomolar range on gelatinases and high selectivity for MMP-2 (see Table 2). The in vivo biodistribution in NMRI white mice and in A549 lung carcinoma bearing athymic mice [78] showed that [^123^I]**27** and [^123^I]**28** have the potential to be used as SPECT tumor imaging agents. 

The biodistribution evaluation in A549-tumor bearing athymic mice showed good tumor uptake for compound [^123^I]**29**, but not so good for compound [^123^I]**30** [79]. A metabolite analysis by plasma HPLC after 2h revealed no degradation of [^123^I]**29**, but compound [^123^I]**30** presented 30% of the metabolites, and 65.4% of the intact compound. However, 91.8% of the accumulated activity in the tumor was produced by the intact compound and only 8.1% by the metabolite. The in vivo evaluation revealed that these MMPIs could have potential as tumor imaging agents, but further research is necessary.

Qi-Huang Zheng et al. reported the synthesis of a series of biphenyl sulfonamide based MMPI radiotracers, [^11^C]**31**–**37**, for the evaluation as potential PET cancer imaging agents [80]. Despite exhibiting strong inhibitory activity on MMP-13, no in vivo imaging data have been reported for these compounds.

The same authors reported the synthesis of [^11^C] labeled MMP inhibitors [^11^C]**38** and [^11^C]**39** for the evaluation as PET cancer imaging agents [81]. Compound *S*-**38** is a potent MMPI for several MMP subtypes with IC_50_ ranging from 0.003 to 7.2 μM. The in vitro biological data showed that both [^11^C]**38** and structurally related compound [^11^C]**9** possessed favorable pharmacokinetics making them eligible for in vivo studies. However, even though [^11^C]**38** had good biodistribution in breast cancer animal models, the tumors were invisible in micro PET imaging studies. 

**Scheme 6:**
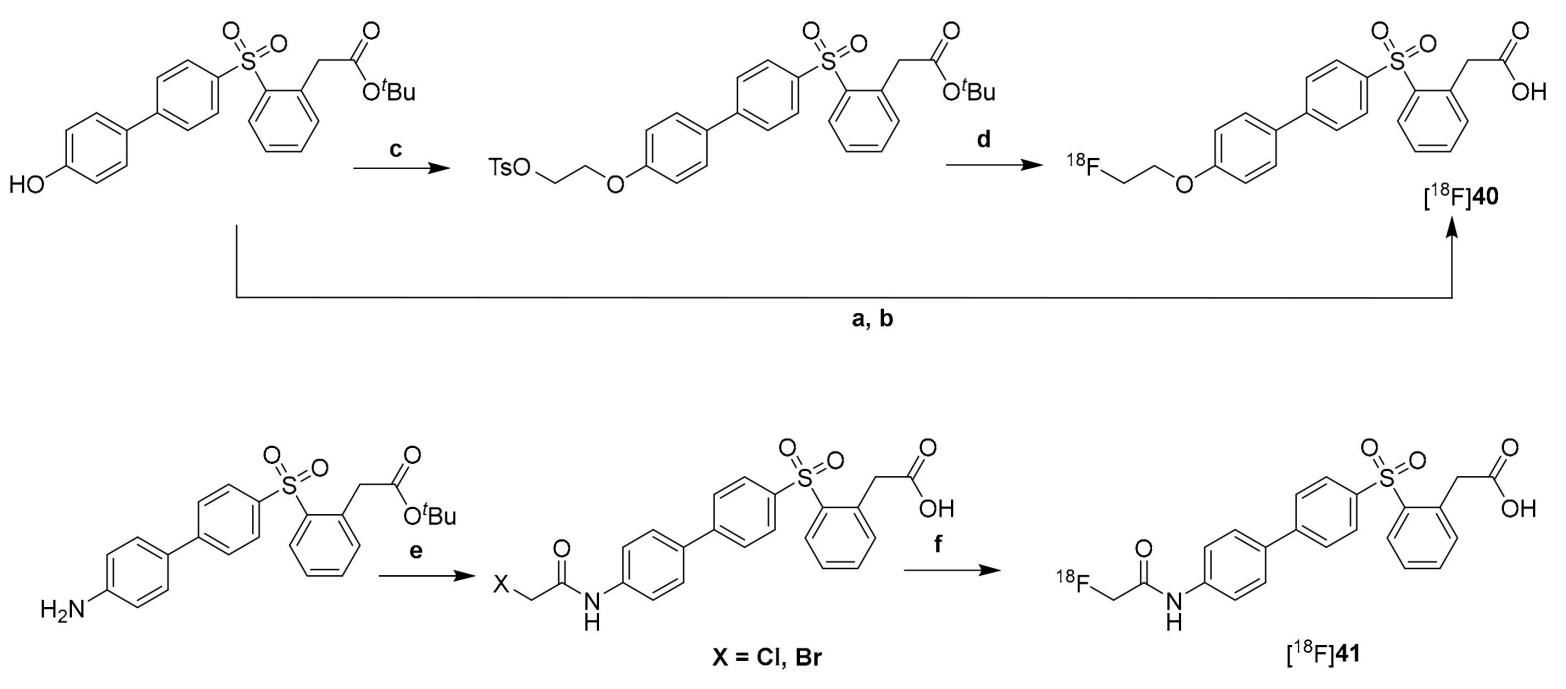
Synthetic route for [^18^F]**40**–**41**. Reagents and conditions: (**a**) (1)C_2_H_4_(OTs)_2_, [^18^F]K(K 222)F, 100 °C; (2) MeONa, 120 °C. (**b**) TFA, CH_3_CN, rt. (**c**) C_2_H_4_(OTs)_2_, K_2_CO_3_, CH_3_CN, 82 °C. (**d**) (1) [^18^F]K(K 222)F, 105 °C; (2) TFA, CH_3_CN, rt. (**e**) Chloroacetylchloride or bromoacetylbromide, DIPEA, DMF. (**f**) (1) [^18^F]K(K 222)F, 130 °C; (2) TFA, CH_3_CN, rt.

Kuhnast et al. reported the synthesis and labelling by methylation with [^11^C]CH_3_I of a *N*-sulfonylamino acid derivative to obtain compound [^11^C]**39** [82]. This compound is a selective and highly potent MMP-2 and MMP-9 inhibitor. Compound [^11^C]**39** exhibited a strong inhibitory effectiveness for gelatinases (110 nM for MMP-2 and 200 nM for MMP-9). In vivo studies in normal mice demonstrated favorable pharmacokinetics and [^11^C]**39** was found to be rapidly excreted and stable up to 30 min post injection. Thus, compound [^11^C]**39** seems to be a suitable compound for PET imaging of MMPs activity. However, the evaluation of this derivate in tumor bearing mice is needed to further validate its applicability. Digilio et al. reported the synthesis of ^18^F labelled arylsulfones [^18^F]**40** and [^18^F]**41** based on the nanomolar activity and selectivity of their non-radioactive counterparts (Scheme 6) [83]. 

**Figure 3:**
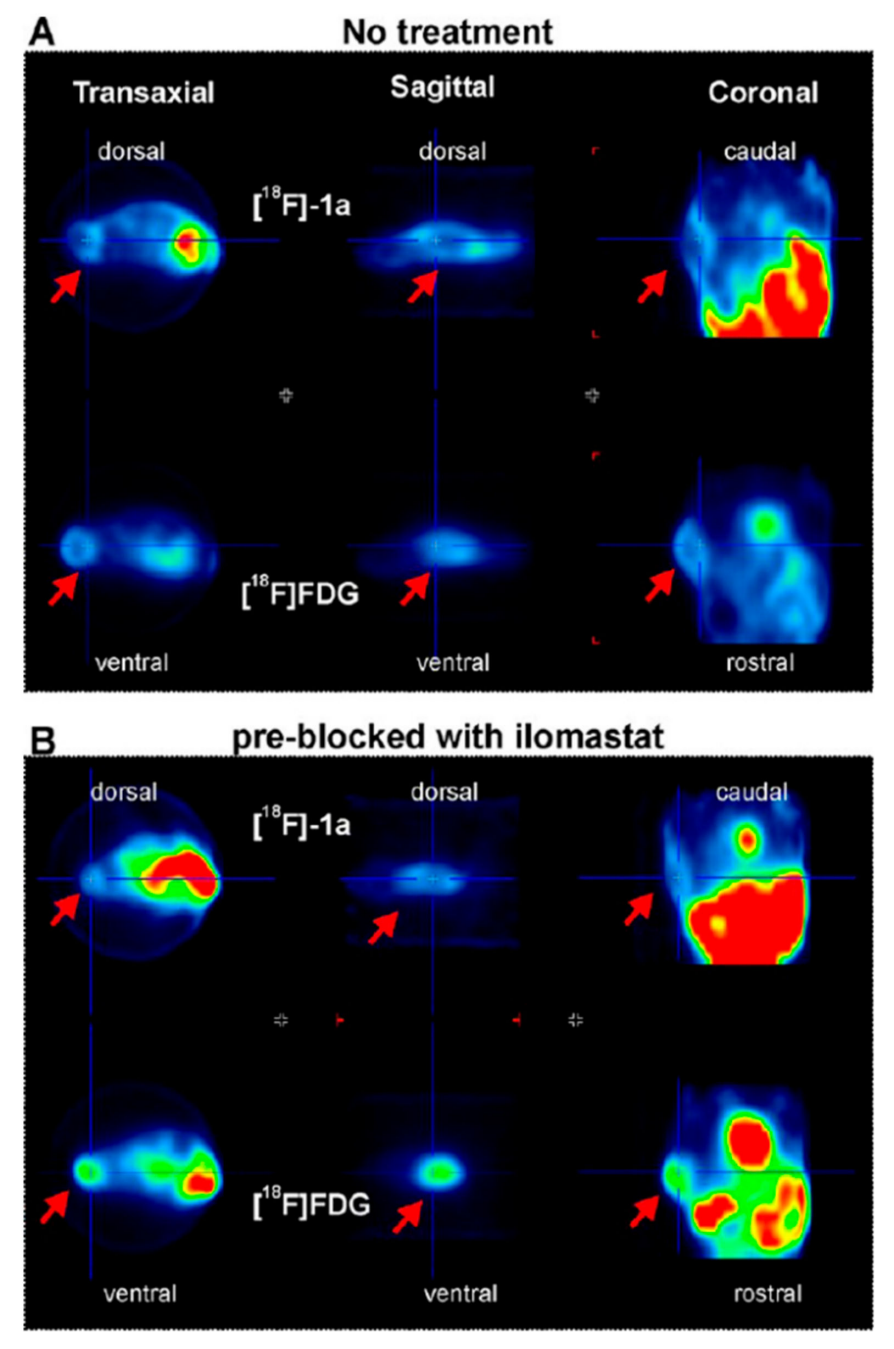
^18^F PET images of the abdomen of nude mice subcutaneously grafted with a human U-87 MG glioblastoma. Red arrows show the tumor in different slices. (**A**) PET images obtained by sequential administration of [^18^F]**40** (**1a**) (upper row) and [^18^F]FDG (bottom row), showing colocalization of tracer uptake. The [^18^F]**40** (**1a**) scan was acquired 100 min post-tracer injection; [^18^F]FDG was injected immediately after completion of the [^18^F]**40** (**1a**) scan and the image was acquired 45 min post-[^18^F]FDG injection. Both scans were static acquisitions (injected dose 7.4 MBq, acquisition time 30 min). (**B**) PET images obtained by sequential administration of [^18^F]**40** (**1a**) (upper row) and [^18^F]FDG (bottom row), in a mouse that received 40 mg/kg of the broad-spectrum MMPI Ilomastat 30 min before injection of [^18^F]**40** (**1a**). The timing scheme and injected doses are the same as in panel A. Reprinted with permission form [83] Copyright © 2013 American Chemical Society.

Tracer [^18^F]**40** was selected to study the uptake of radioactivity in a human glioblastoma mouse model as this compound presented the best MMP inhibition profile and efficiency of radiosynthesis. The biodistribution of [^18^F]**40** was dominated by liver uptake, gastrointestinal tract uptake and hepatobiliary clearance. When compared to [^18^F]fluorodeoxyglucose (FDG), compound [^18^F]**40** exhibited an excellent tumor uptake and demonstrated to be specific (Figure 3).

### 2.5. Barbiturate Based MMPIs Labelled Imaging Probes

**Scheme 7:**
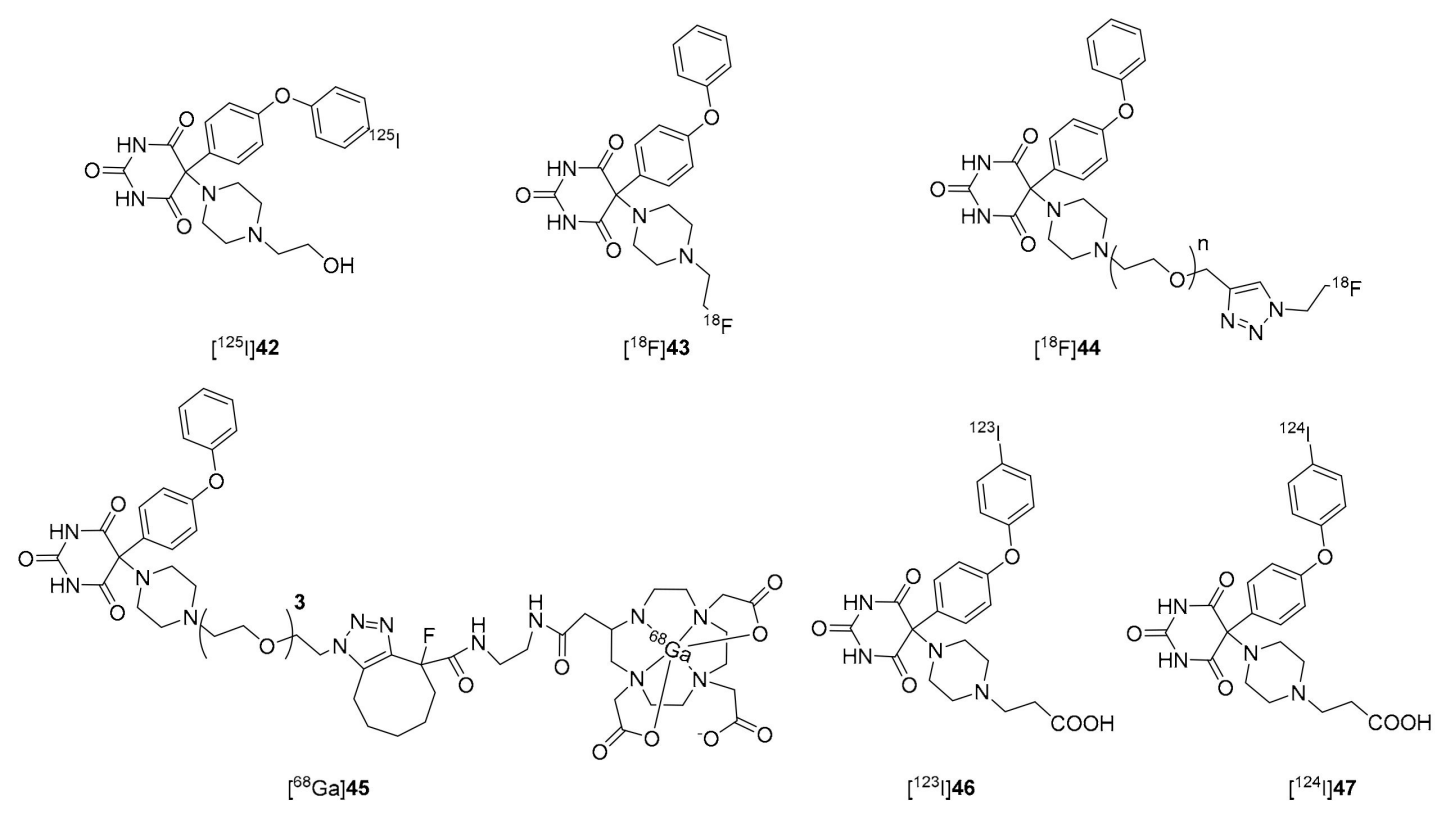
Chemical structure for compounds **42**–**47**.

Breyholz et al. described a barbiturate-based MMPI radiotracer [^125^I]**42** (Scheme 7) for the noninvasive in vivo visualization of activated MMPs using SPECT or PET imaging modalities [84]. The corresponding non-labeled compound showed some selectivity towards MMP-2 and -9 (IC_50_ MMP-2 = 7 nM; MMP-9 = 2 nM).

The same group reported the structurally related [^18^F]43 radiotracer [85]. The unlabeled analogous showed nanomolar MMP inhibition potencies with higher selectivity for MMP-9 against MMP-2 (MMP-2, IC_50_ = 23 nM; MMP-9, IC_50_ = 7 nM). Preliminary biodistribution studies indicated that there was no tissue specific accumulation in WT C57/BL6 mice and demonstrated favorable pharmacokinetic behavior corresponding to the physical half-life of the positron emitter. Small-animal PET images of the coronal whole-body showed that the tracer uptake in non-target organs was low over all the time points and showed rapid clearance through the renal and hepatobiliary route. Thus, these barbiturate-based MMP-targeted radiotracers may be applicable in noninvasive in vivo imaging of MMP-2 and MMP-9-associated diseases by means of PET.

**Scheme 8:**
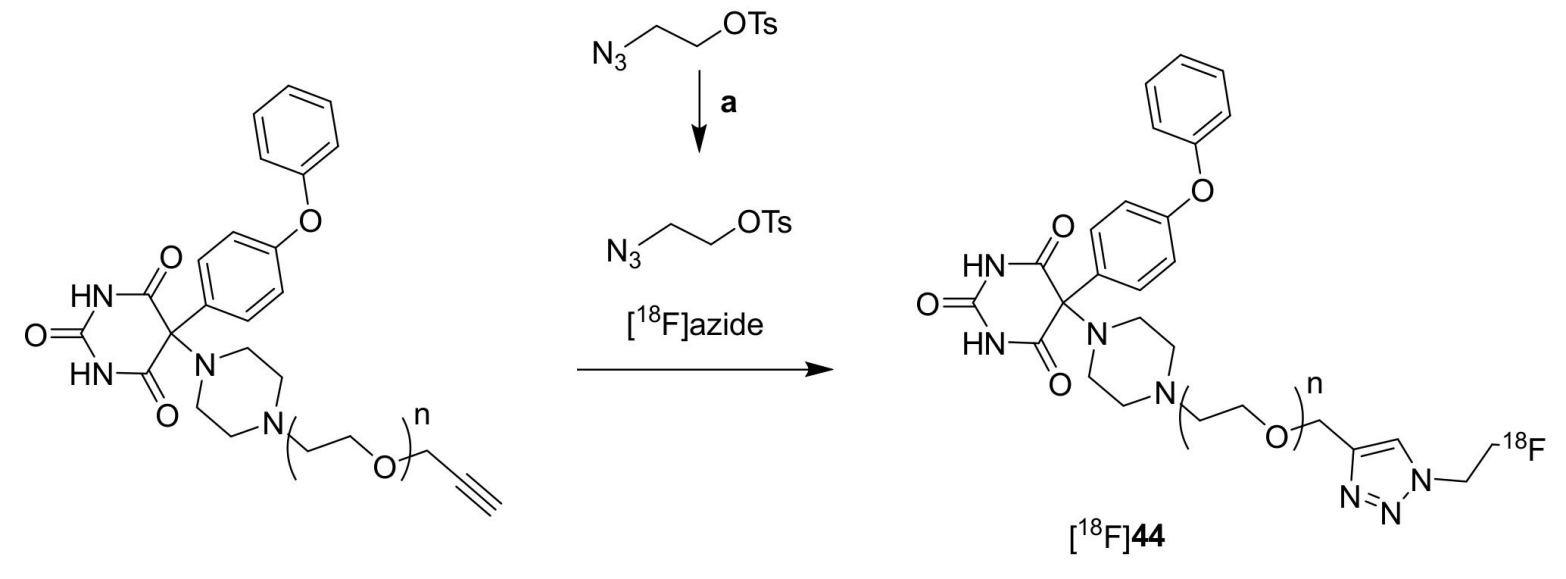
Synthetic route for [^18^F]**44**. Reaction conditions: (**a**) [^18^F]fluoride (potassium 222 cryptate), K_2_CO_3_, CH_3_CN, 84 °C, 15 min; (**b**) CuSO_4_·H_2_O, sodium ascorbate, DMF, rt, 8 min.

A second generation of radio fluorinated barbiturate-based MMP tracers containing a triazole linker was described (Scheme 8) [86]. 

The in vivo biodistribution studies of [^18^F]**44** in WT mice showed a high level of radioactivity in the liver, and a slower liver clearance and faster kidney clearance than the more hydrophobic [^18^F]**43**. Hence, the alteration of the chemical structure by introducing a mini-PEG and a triazole unit in [^18^F]**44** led to a favorable pharmacokinetic behavior for in vivo PET-imaging. However, preclinical PET/CT studies in disease models with known MMP up-regulation (e.g., tumor, Lewis lung carcinoma bearing mice; atherosclerotic plaques, apolipoprotein E-deficient mice) should be assessed [87]. There are also examples of barbiturate MMP inhibitors labelled with [^68^Ga]**45** [88].

More recently Wagner et al. developed the more hydrophilic radioiodinated barbiturate-based MMP tracers [^123^I]**46**-[^124^I]**47**. These compounds presented high inhibitory potency (IC_50_ (MMP-2) = 29 nM, IC_50_ (MMP-9) = 1.3 nM) [88]. 

**Scheme 9:**
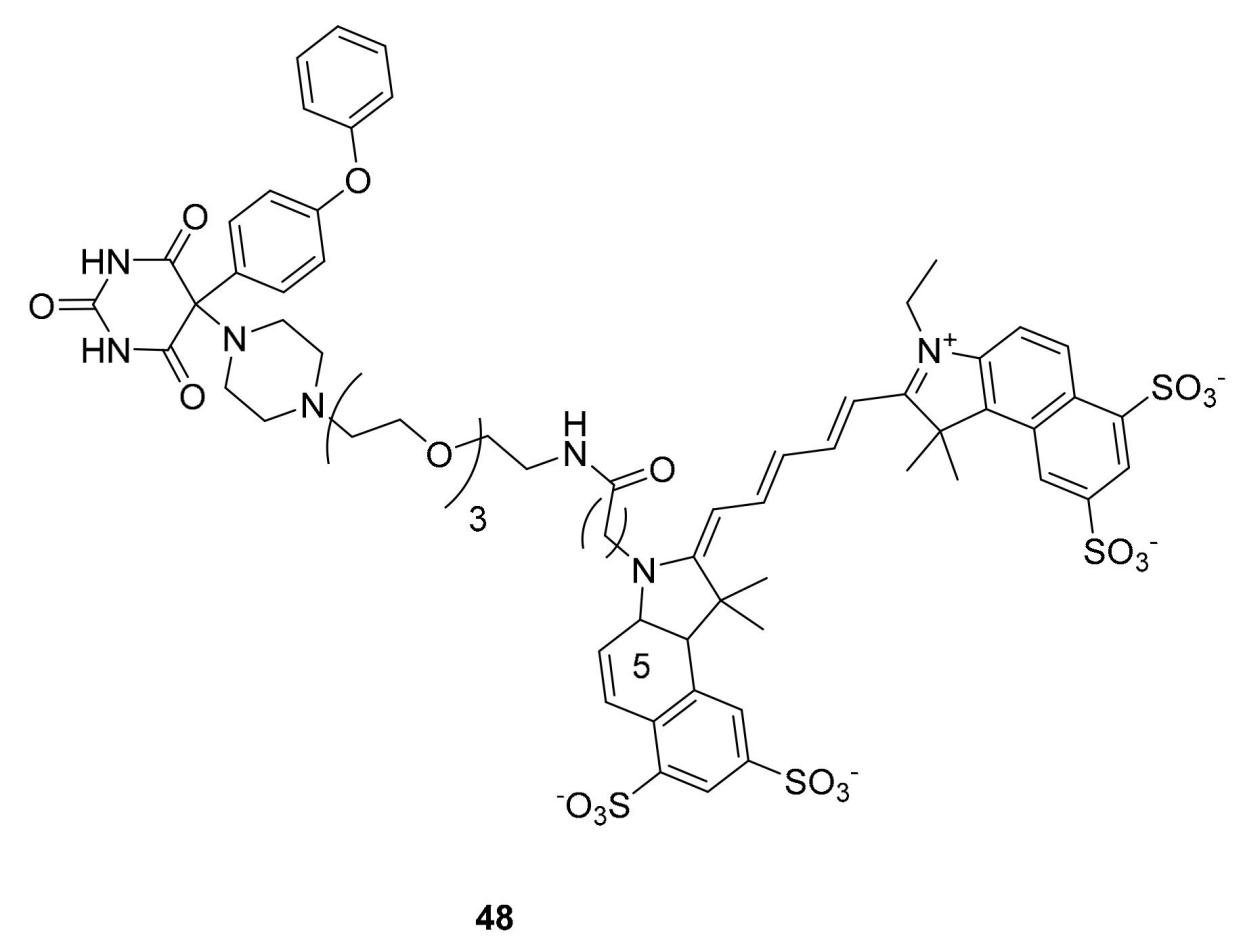
Chemical structure for compound **48**.

In 2008, Faust et al. reported Cy5.5 labeled barbiturate based MMPIs as fluorescence photoprobes for imaging MMPs [89]. They designed a convergent 10 step synthetic route to obtain non-hydroxamate MMP inhibitor **48**, which showed high affinity (IC_50_ value: 48 nM for MMP-2) measured by a fluorogenic assay using commercially available MMP-substrates and the purified enzyme. Fluorescence microscopy studies using highly (A-673, rhabdomyosarcoma) and moderate (HT-1080, fibrosarcoma) MMP-2 expressing cell lines showed efficient binding of **48** to the MMP-2 positive cells while no binding to MMP-2 negative cells (MCF-7, breast cancer cells). Pre-dosing experiments using 50 μM of the unlabeled barbiturate successfully blocked the binding of the Cy 5.5-labeled ligand **48** and showed specific uptake of the tracer. Therefore Cy 5.5 labeled tracer **48** (Scheme 9) may be a promising candidate for sensitive MMP detection in vivo. 

### 2.6. Marimastat Based MMPIs Labelled Imaging Probes

**Scheme 10:**
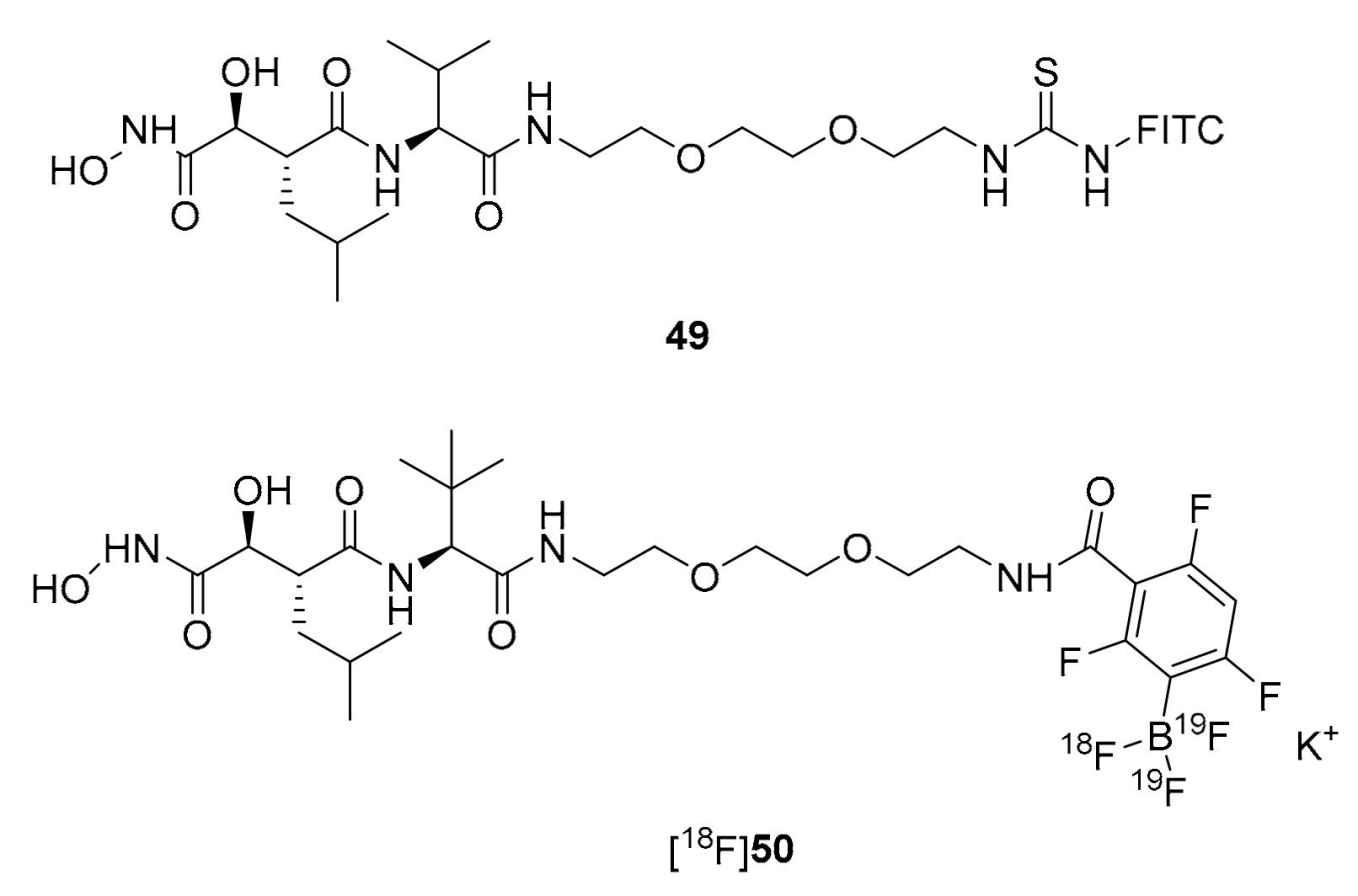
Chemical structure for compounds **49**–**50**.

Overall, a series of labelled Marimastat derivatives for in vivo PET imaging of MMP in cancer [90,91] have been described. First, FITC conjugated Marimastat (**49**) (Scheme 10) was reported to image MDA-MB-231 breast cancer cells stably transfected with MMP-14 by fluorescence. 

Later, a one-step synthesis of stable [^18^F]aryltrifluoroborate-**50** under mild conditions by the use of a stable arylboronic acid as a captor of aqueous [^18^F]fluoride was reported. This facile method avoids tedious multistep syntheses or harsh fluorination conditions. 

Syngeneic 67NR murine mammary carcinoma or 67NR/CMV luciferase derived primary tumor in Balb/C mice were used for in vivo PET imaging. After the injection of [^18^F]**50** to tumor-bearing mice and PET image acquisition, a low but detectable and specific uptake in the primary tumor was observed. However, in mice injected with higher concentration of [^18^F]**50** or control-ArBF_3_ lacking the Marimastat moiety, no tumor localization was observed, with clearance only to the liver, bladder, and submaxillary salivary gland. As a specificity control, tumor-bearing mice were blocked with an unlabeled marimastat prior to the tracer injection. The pre-blocked mice had clearly reduced uptake with similarly sized tumors. The time-activity curve analysis revealed that [^18^F]**50** accumulated in the tumor after 60 min. Overall, the PET imaging and the time-activity curve analyses showed specific ^18^F-labeling of the tumor as well as the bladder, liver, stomach, and gut.

### 2.7. Tripeptide Hydroxamic Acid Labelled with ^18^F as Imaging Probes for MMPs

**Scheme 11:**
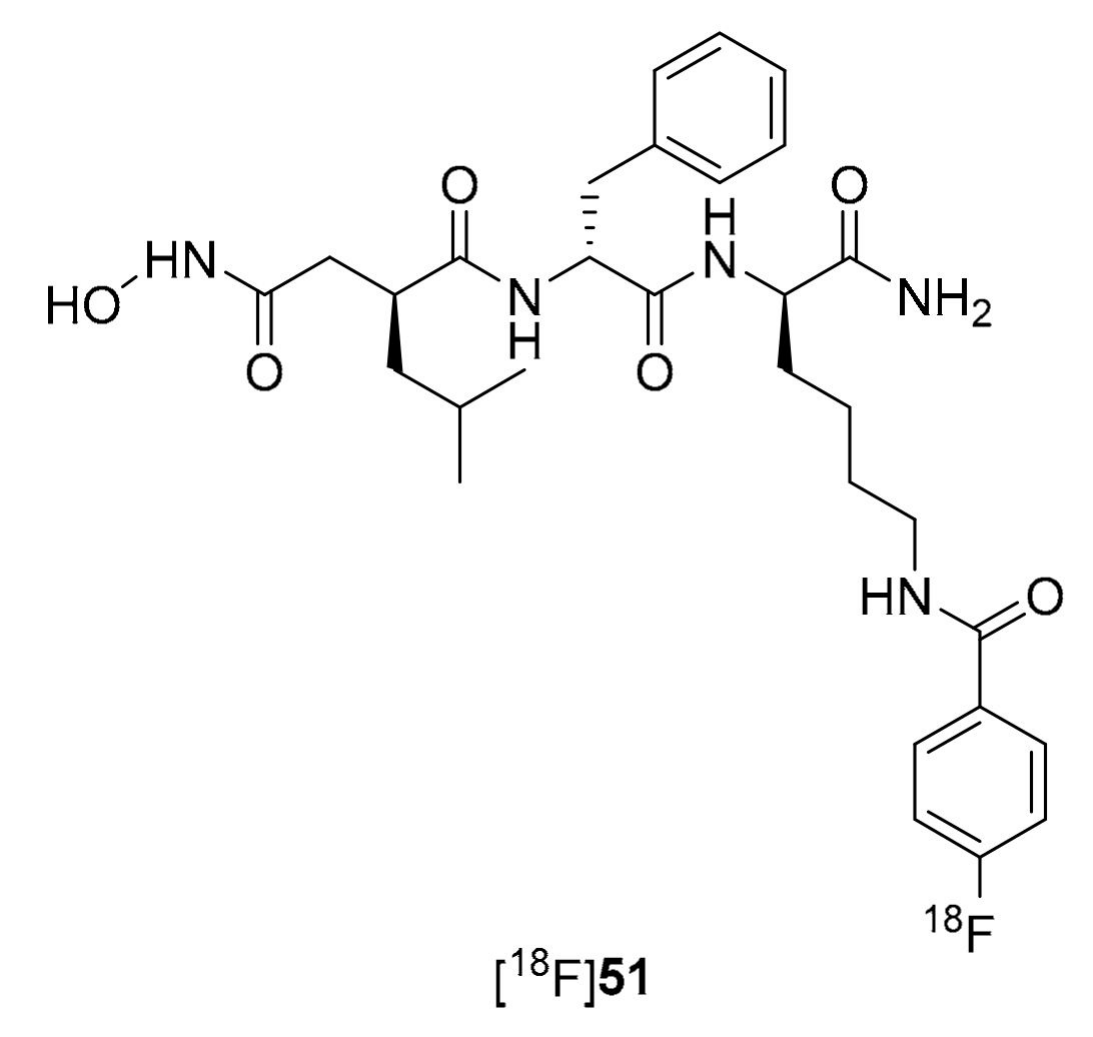
Chemical structure for compound **51**.

Elsinga and colleagues synthesized [^18^F]**51** (Scheme 11) by the direct acylation of the corresponding unlabeled inhibitor with *N*-succinimidyl-4-[^18^F]fluorobenzoate [92]. 

The resulting radiotracer [^18^F]**51** was evaluated in human bronchial epithelium 16HBE cells and breast cancer MCF-7 cells showing rather low binding to these cell lines. Specificity studies showed that cellular binding was reduced by 36.6% and 27.5% in MCF-7 and 16HBE cells, respectively, after co-incubation with the unlabeled counterpart.

The in vivo kinetics of [^18^F]**51** were examined in a HT1080 fibrosarcoma tumor bearing mice. In microPET scans, HT1080 tumors exhibited a low and homogeneous uptake of the tracer, suggesting that tracer binding was not only to membrane-bound disintegrin and metalloproteinase (ADAMs) but also to the extracellular MMPs. The tumors of mice injected with [^18^F]**51** showed specific uptake confirmed by blocking studies. The ex vivo biodistribution showed a rapid excretion through the kidneys and the liver. The metabolite assays indicated that the parent tracer represented 23.2 ± 7.3% (*n* = 2) of the total radioactivity in plasma after one and half hours after treatment. The autoradiography of a tumor slice confirmed the regular uptake on the tumor. A high kidney uptake was observed in the microPET/CT images. The change of the tumor to plasma ratio was statistically significant in contrast to the change of the tumor to muscle ratio. Therefore, the binding of [^18^F]**51** in the HT1080 xenograft mice was target mediated. Compound [^18^F]**51** may be suitable for the visualization/quantification of diseases overexpressing simultaneously MMPs and ADAMs.

### 2.8. Thiirane Based MMPIs Labelled Imaging Probes

**Scheme 12:**
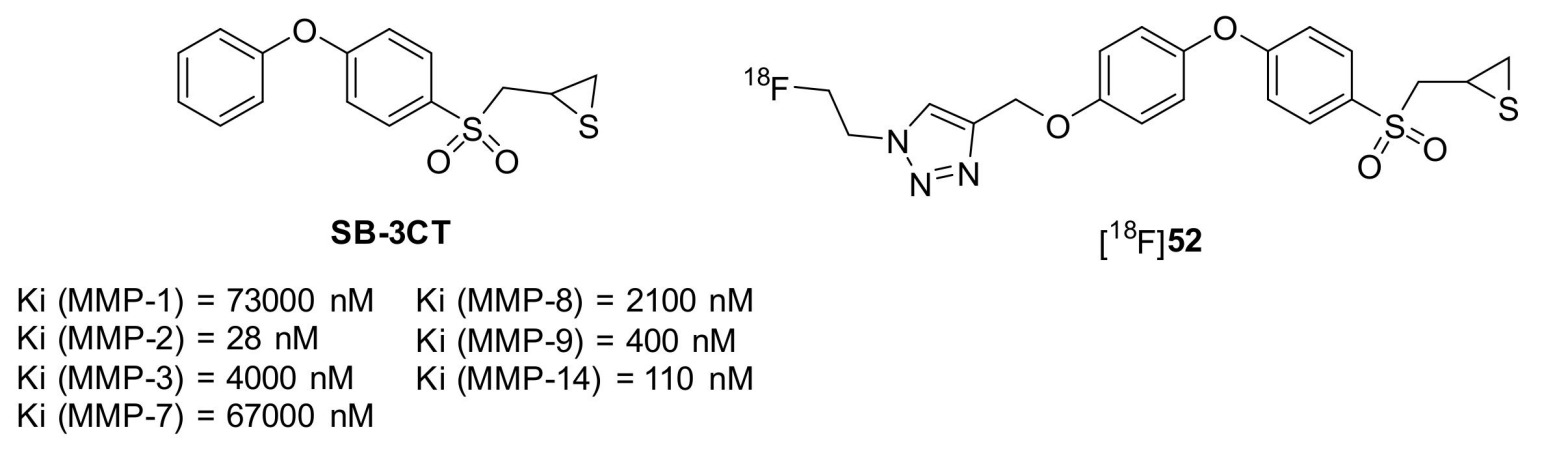
Chemical structure for compounds SB-3CT and [^18^F]**52** and *K_i_* values against several MMPs.

Wagner et al. reported ^18^F-fluorine labeled radiotracers based on SB-3CT, a slow-binding and mechanism-based MMP-2 and -9 inhibitor [93]. Inspired by the selectivity and activity of SB-3CT, they synthesized non-radiolabeled analogues and evaluated their MMP inhibition profile showing *K*_i_ values ranging from 20.9 nM to 815 nM. These values are comparable to SB-3CT but they were non-selective inhibitors, with mixed kinetics and attractive residence times for MMP-2. However, this compound was unstable in human and mouse serum. The in vivo biodistribution studies revealed a rapid hepatobiliary elimination of the thiirane [^18^F]**52** (Scheme 12) and its metabolites in C57BL/6 mice and therefore, it is not suitable for PET imaging and further in vivo evaluations in MMP associated mouse models of disease.

## 3. MMPIs Labelled Imaging Agents for Atherosclerosis, Myocardial Infarction and Aneurysm

### 3.1. CGS27023A Based MMPIs Labelled Imaging Probes

CGS27023 is a broad spectrum MMP inhibitor used by Schafers et al. to develop [^123^I]**53** for in vivo imaging of MMP activity [94], and by Hartung et al. for the development of [^125^I]**54** and [^124^I]**55** for autoradiography and PET studies in apoE^−/−^ mice, respectively [95]. 

The cold compound **53** yielded nanomolar IC_50_ values for MMP inhibition (298 and 153 nM for MMP-2 and MMP-9, respectively) making it suitable for radioligand synthesis for in vivo imaging of these MMPs activities. Upon pretreatment with non-labeled compound **53**, compound [^123^I]**53** was able to specifically image MMP activity in vivo in the MMP-rich vascular lesions that develop after carotid artery ligation and a cholesterol-rich diet in apolipoprotein E-deficient mice, using WT C57/BL6 mice as the control. Additionally, compound [^123^I]**53** presented no uptake in thoracic cavity and the brain and was mainly excreted through the renal and hepatic pathways.

**Figure 4:**
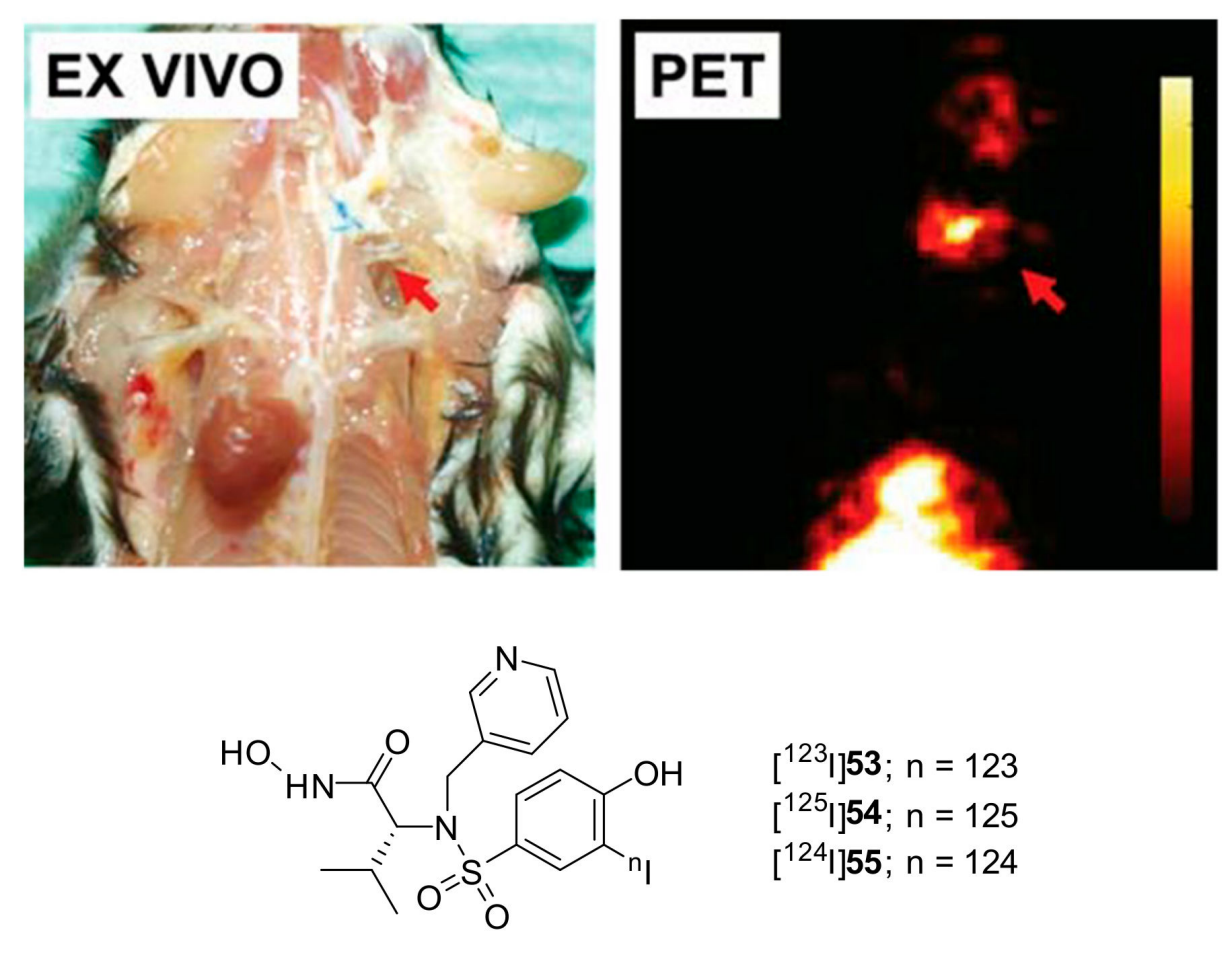
The site of the ligated left common carotid artery (left panel) and a corresponding whole-body coronal slice (0.4 mm thick) through a left carotid lesion (right panel) 4 weeks after ligation and a HC diet in an apoE−/− mouse. The intense uptake of the radiolabeled broad spectrum MMP inhibitor [^124^I]**55** in the left carotid lesion (arrow) 30 min after intravenous injection is visible using high-resolution small animal PET. Reprinted with permission from ref [95] Copyright © Springer-Verlag 2007.

Compound [^125^I]**54** presented no specific uptake in the ligated carotid arteries and the results did not improve upon pretreatment with the non–labeled compound. Compound [^124^I]**55** presented a specific uptake in the ligated carotid arteries which was significantly higher in mice injected with [^124^I]**55** alone than in mice pretreated with an excess of unlabeled MMPI (Figure 4). 

Although additional studies are needed to test the potential of this approach as a novel noninvasive clinical diagnostic tool for the management of human MMP-related cardiovascular diseases, these results prove that imaging of MMP activity in vivo is feasible using radiolabeled MMPIs in vascular lesions.

### 3.2. Macrocyclic Hydroxamate Based MMPIs Labelled Imaging Probes

**Scheme 13:**
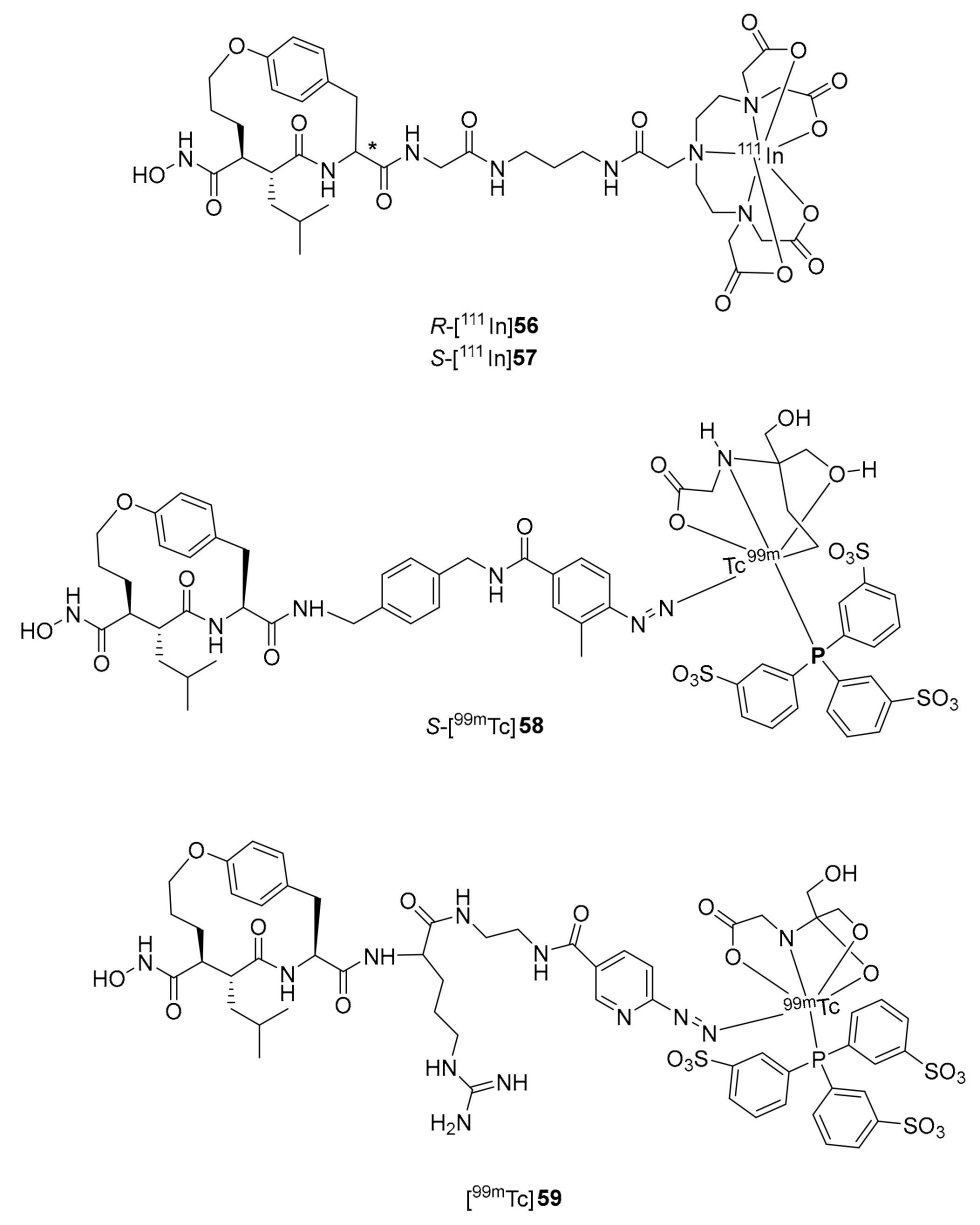
Chemical structure for compounds **56**–**59**.

The activation of MMPs after myocardial infarction (MI) contributes to adverse left ventricular (LV) remodeling. This remodeling process gives rise to LV dysfunction and progressive heart failure. The radiolabeled compounds [^111^In]**56**, [^111^In]**57** and [^99m^Tc]**58** were studied by Sinusas et al. [96], while Narula et al. developed the labelled tracer [^99m^Tc]**59** (Scheme 13) [97].

**Figure 5:**
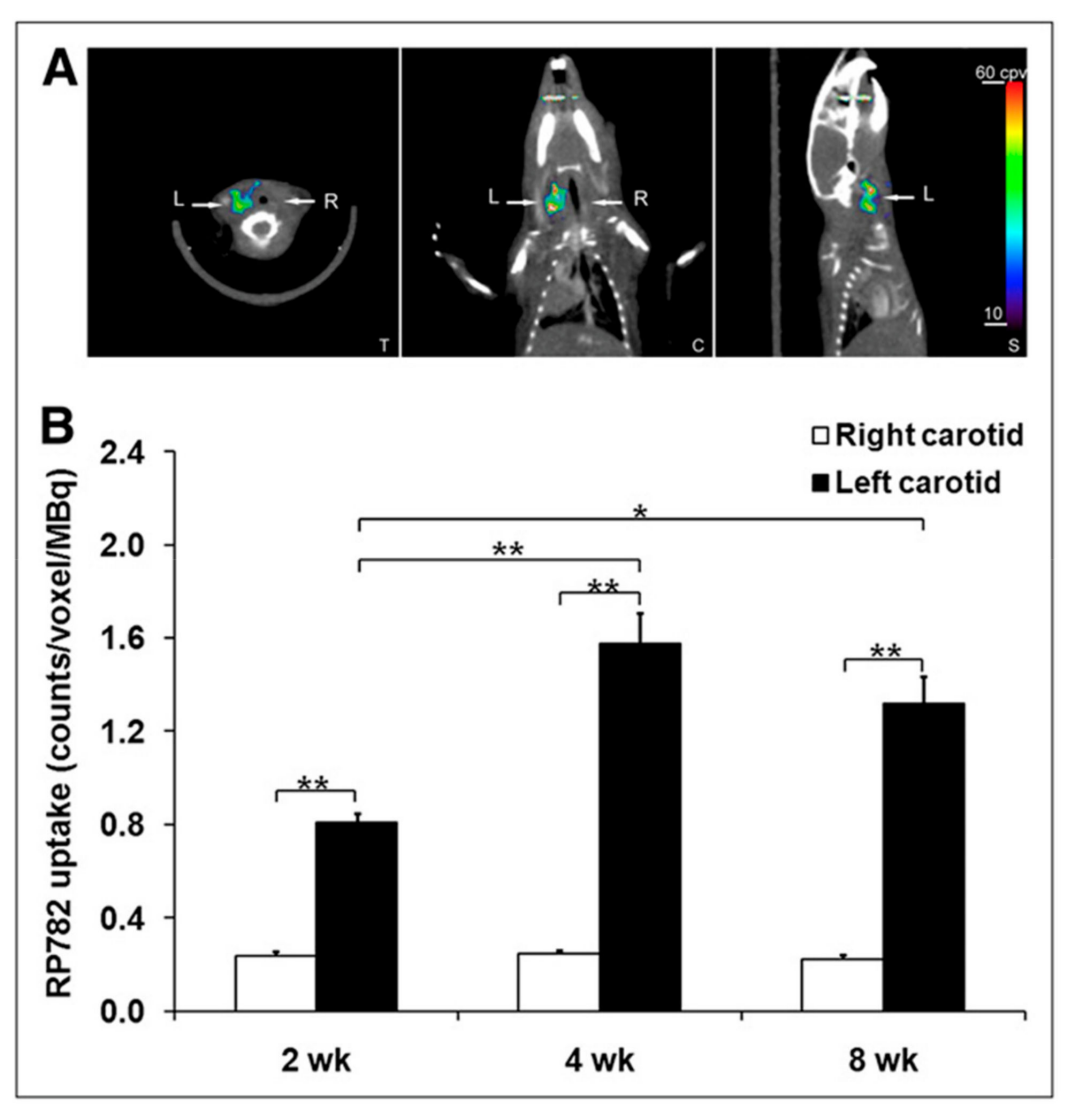
In vivo imaging of MMP activation in aneurysm. (**A**) An example of fused micro SPECT/CT images of mouse at 4 weeks after surgery to induce carotid aneurysm. The arrows point to aneurysmal left (L) and control right (R) carotid arteries. (**B**) The image-derived quantitative analysis of background-corrected [^111^In]**56** (RP782) carotid uptake. The background-corrected tracer uptake in left carotid artery peaked at 4 weeks after surgery and was significantly higher than uptake in right carotid artery at every time point studied. n 5 16-18 in each group. * *p* = 0.01. ** *p* < 0.001. C 5 coronal slice; S 5 sagittal slice; T 5 transverse slice. Reprinted with permission from [98] copyright © 2010 by the Society of Nuclear Medicine, Inc.

[^111^In]**56** showed specific uptake by microautoradiography in surgically induced MI in C57BL/6 mice, using [^111^In]**57** as a negative control. Further, gamma well counting demonstrated a 3- to 4-fold increase uptake in the same animal model. The same authors demonstrated, by using a model of carotid aneurysm in apolipoprotein E deficient mice, that *R*-[^111^In]**56** is a useful radiotracer to noninvasively predict the propensity of an aneurysm to expand (Figure 5) [98].

**Figure 6:**
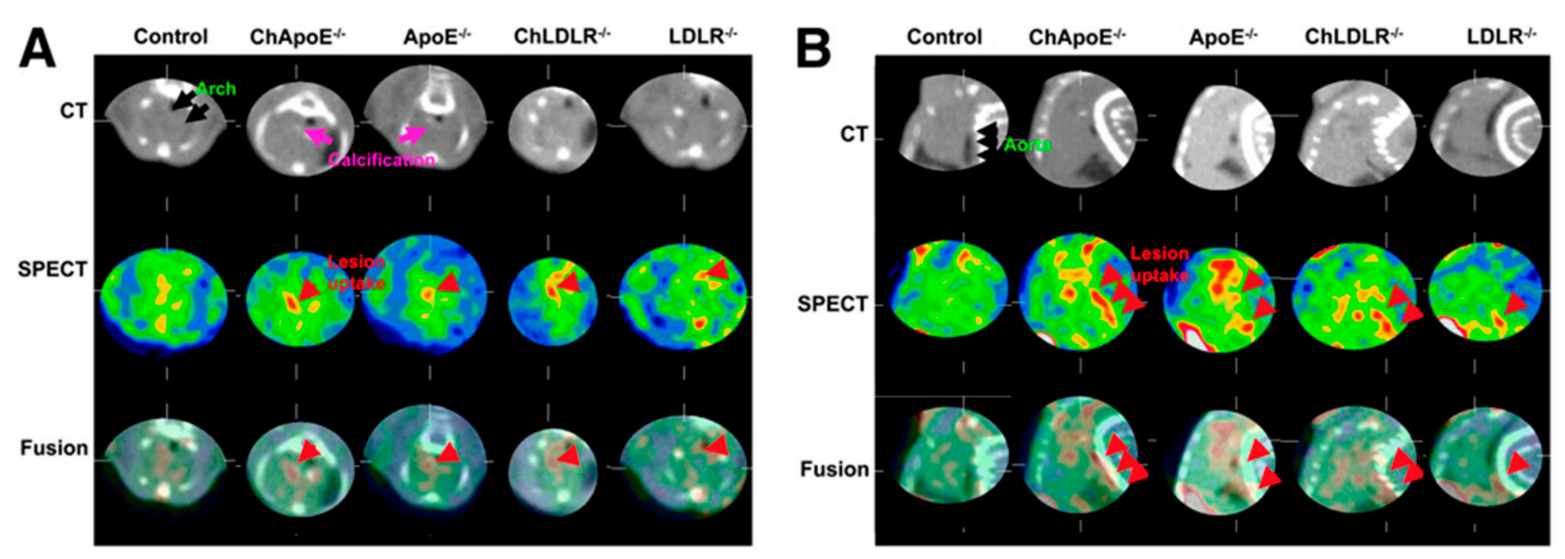
In vivo micro-SPECT/ micro-CT transverse (**A**) and sagittal (**B**) zoomed and masked images of the 5 study groups. Compound [^99m^Tc]**58** uptake in atherosclerotic aortic lesions was identified noninvasively in arch and abdominal aorta by micro-SPECT aided by micro-CT; aortic arch calcification was detected by micro-CT. Ch 5 high-cholesterol-fed. Reprinted with permission from [97] copyright © 2009 by the Society of Nuclear Medicine, Inc.

**Figure 7:**
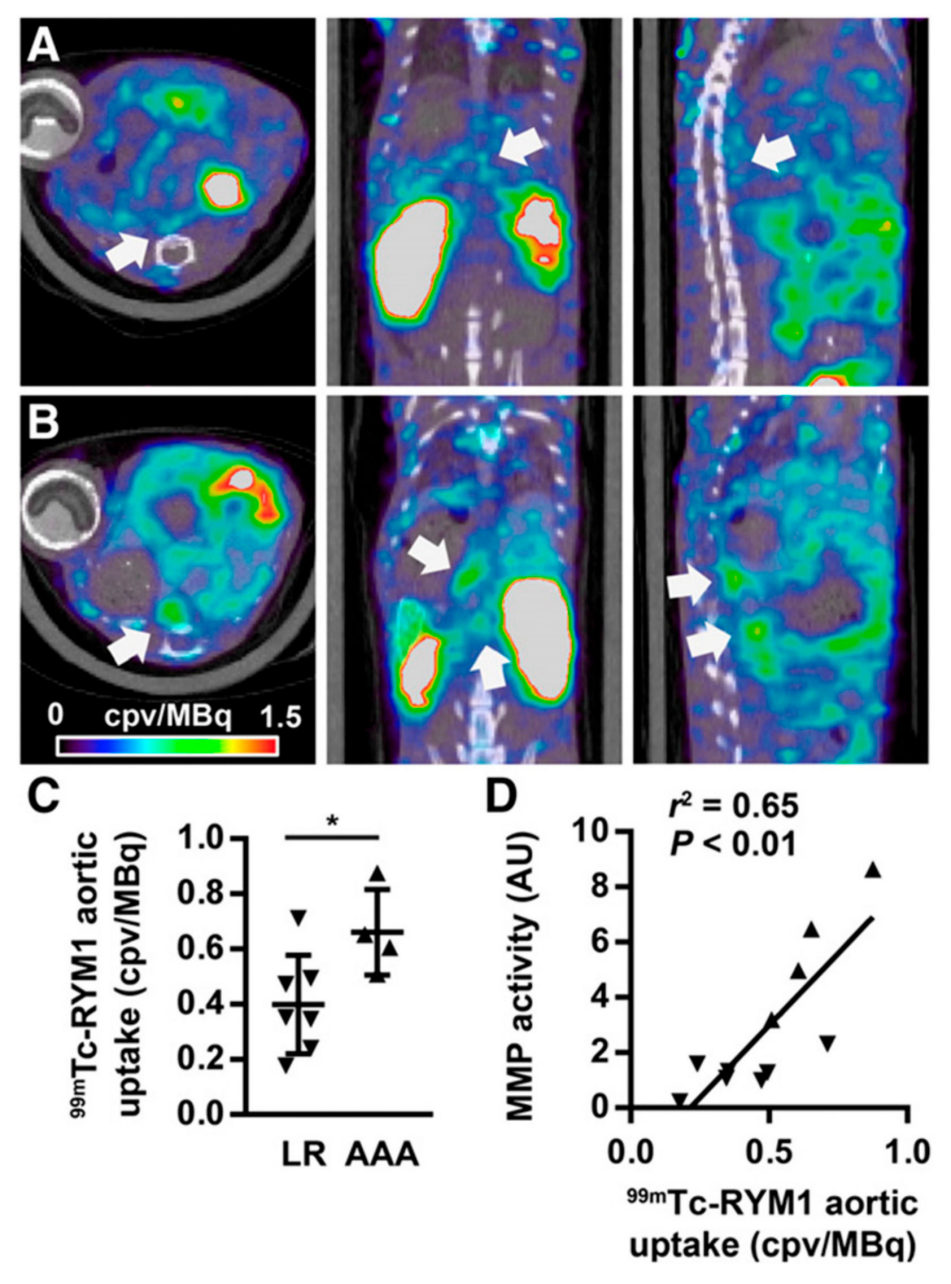
Compound [^99m^Tc]**59** (RYM1) imaging of AAA. (**A**,**B**) Examples of fused [^99m^Tc]**59** (RYM1) SPECT/CT images of animals from the low remodeling (**A**) and aneurysm (**B**) groups, classified on the basis of a visual in situ analysis of abdominal aorta. Transversal (left), coronal (middle), and sagittal (right) views are shown. Arrows point to areas of maximal tracer uptake in abdominal aorta. (**C**) Quantification of [^99m^Tc]**59** (RYM1) signal in area of maximal tracer uptake in suprarenal abdominal aorta in low remodeling and AAA groups. * *p* < 0.05. (**D**) Correlation between [^99m^Tc]**59** (RYM1) signal in vivo and MMP activity quantified by zymography ex vivo. AU 5 arbitrary units; cpv 5 counts per voxel; LR 5 low remodeling. Reprinted with permission from [99] copyright © 2017 by the Society of Nuclear Medicine and Molecular Imaging.

The micro-SPECT/CT imaging studies with analogous [^99m^Tc]**58** radiotracer demonstrated a 5-fold increase of myocardial uptake. Additionally, tracer [^99m^Tc]**58** showed specific uptake in atherosclerotic lesions in apolipoprotein E (ApoE^−/−^) mice deficient in the low-density-lipoprotein receptor (LDLR^−/−^) (Figure 6). After treating abdominal aortic aneurysm (AAA) mouse models with [^99m^Tc]**59**, the SPECT/CT images showed that [^99m^Tc]**59** displayed a maximum tracer uptake (Figure 7). Although further validation is required, [^99m^Tc]**59**-based molecular imaging might improve patient AAA management, as well as other disorders associated with MMP activity dysregulation.

### 3.3. N-Sulfonylamino Acid Based Mmpis Labelled Imaging Probes

**Scheme 14:**
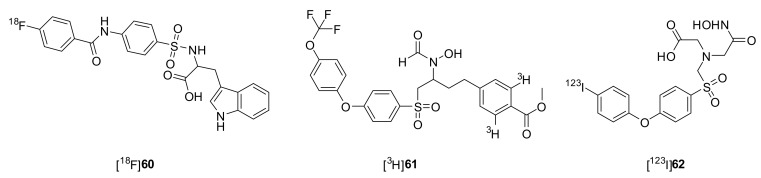
Chemical structure for compounds **60**–**62**.

Radiotracers [^18^F]**60**, [^3^H]**61** and [^123^I]**62** (Scheme 14) have been studied by Selivanova et al. [100], Müller et al. [101] and Windhorst et al. [102] for the detection of MMPs in human atherosclerosis plaques. Both compounds [^18^F]**60** and [^3^H]**61** were evaluated by in vitro autoradiography which showed that they presented specific uptake in vulnerable atherosclerotic plaques. 

[^123^I]**62** presented a high selectivity for MMP-2/-9 over MMP-1. The biodistribution and autoradiography studies of [^123^I]**62** showed an uptake in atheroprone mice, which is a clear indicator of the suitability of this compound for SPECT imaging. However, metabolite detection studies should be carried out.

## 4. MMPIs Labelled Imaging Agents for Experimental Autoimmune Encephalomyelitis (EAE) and Multiple Sclerosis (MS)

MMP-2 and MMP-9 are important for the induction of neuroinflammatory symptoms in experimental autoimmune encephalomyelitis (EAE) in a multiple sclerosis (MS) mouse model [103]. 

**Figure 8:**
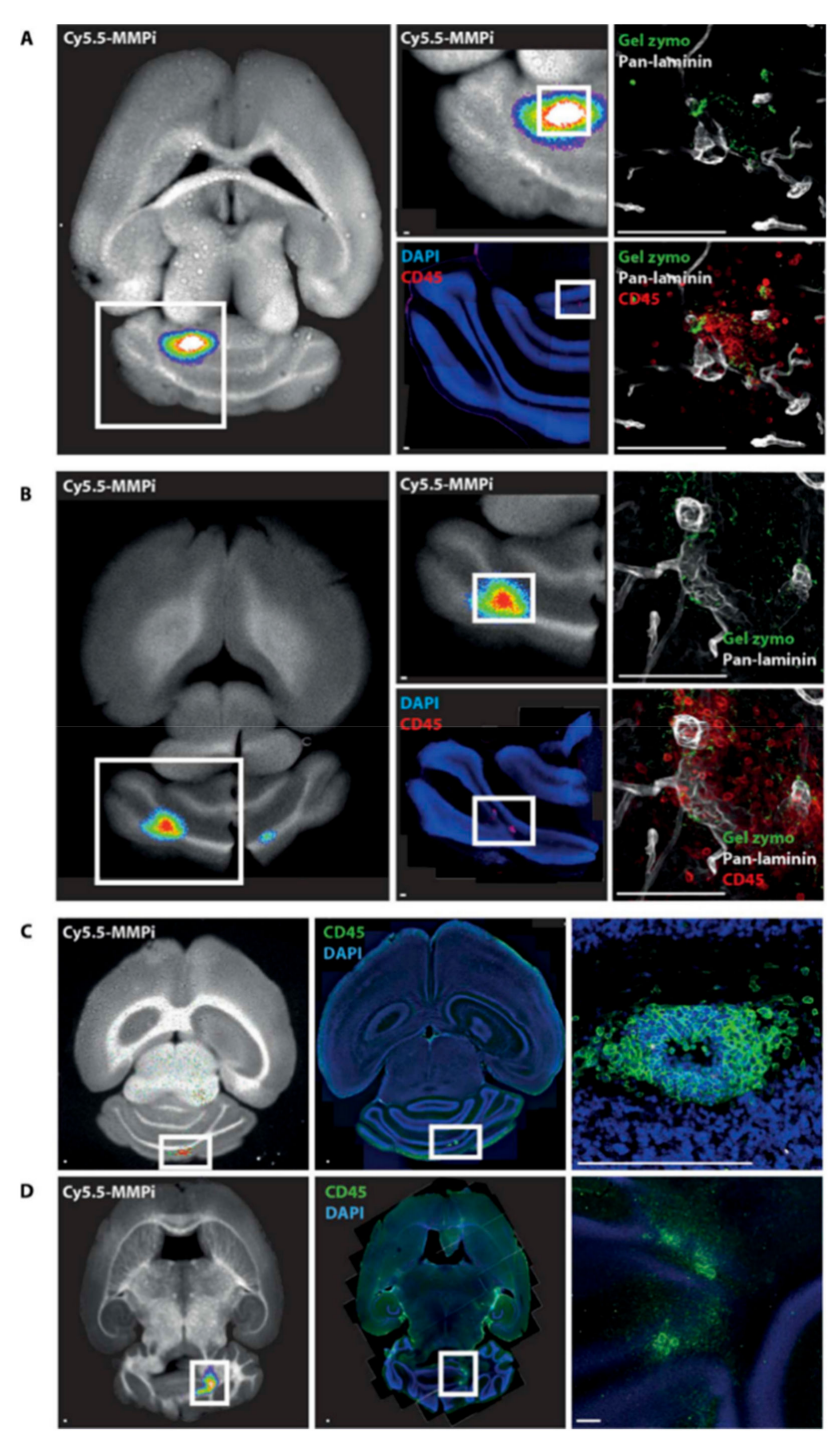
Cy5.5-MMPI **21** tracer signal localizes at sites of perivascular cuffs and gelatinase activity in vivo. (**A**,**B**) Five hours after intravenous injection of **21** (4 nmol) in two score 3 EAE mice, the brains were sliced into thick sections (200 mm) and imaged by FRI; color-encoded near infrared fluorescence and white-light images were captured and matched (left images). The same brain slices were then analyzed by in situ zymography for detection of activated gelatinases and subsequently stained with pan-laminin to define vessel borders and with CD45 antibodies to visualize inflammatory infiltrates (right images). Scale bars, 100 mm. (**C**,**D**) FRI of thick EAE brain sections (200 mm) at 5 h after intravenous injection of (4 nmol). Overlay of color-encoded near-infrared fluorescence image with significant MMP activity (red) projected onto the white-light image. The same brain slice was subsequently immunofluorescently stained with CD45 antibody; blue signal. 4′,6-diamidino-2- phenylindole (DAPI) staining of nuclei. Two separate brains are shown in (**C**,**D**). Scale bars, 100 mm. Reprinted with permission from [103] Copyright © 2016, American Association for the Advancement of Science.

Compound **21** and [^18^F]**15**, which were mentioned before as cancer probes, were also studied for EAE and MS. The injection of **21** in EAE mice showed an uptake at higher severities. Blocking studies demonstrated its specific uptake in EAE tissues (Figure 8). 

**Figure 9:**
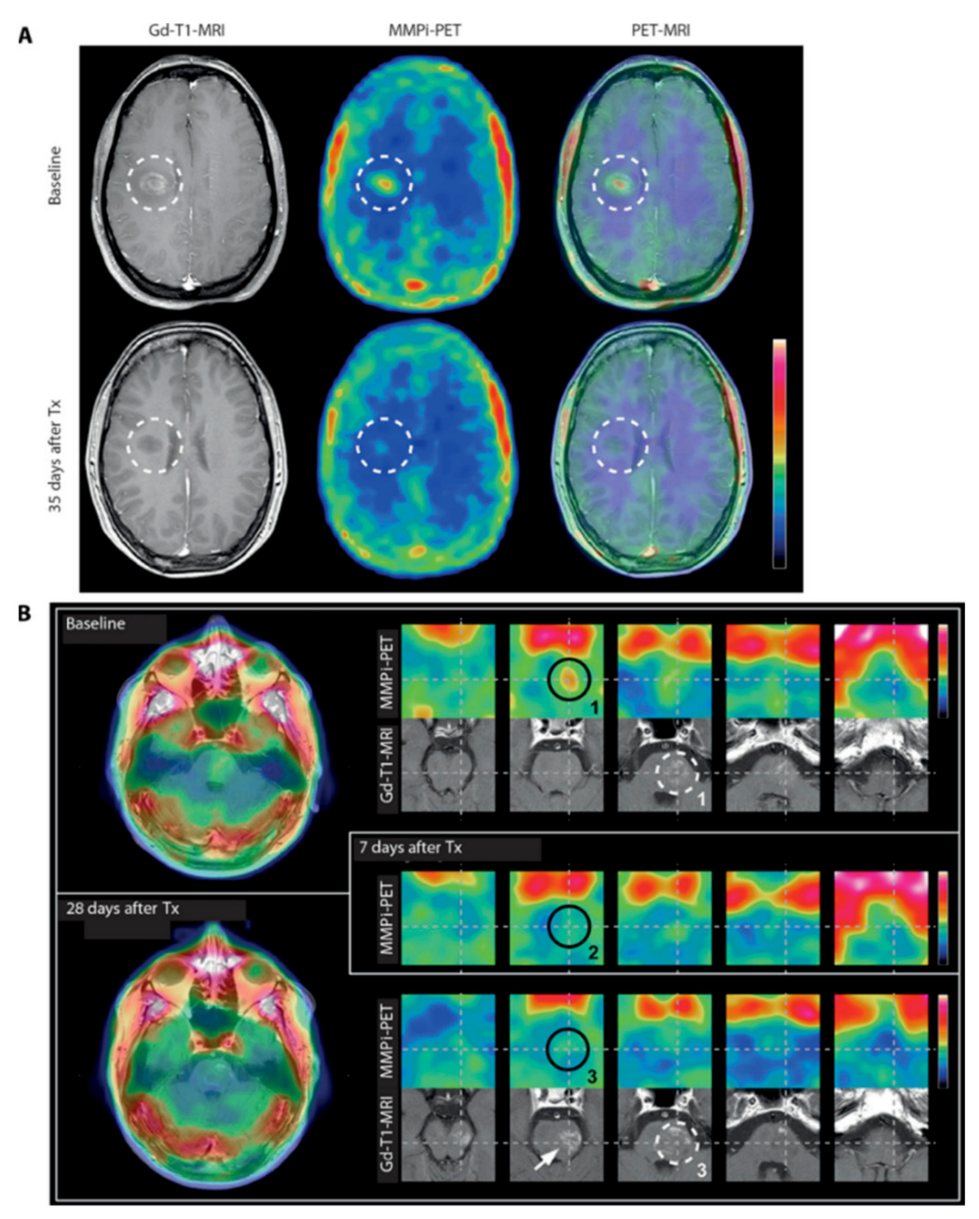
MMPI-PET [^18^F]**15** and Gd-T1-MRI in MS patients before and after anti-inflammatory treatment. (**A**) [^18^F]**15** and Gd-T1-MRI of patient #6 representing an acute attack of RRMS (EDSS 2.0) at baseline (top) and at 35 days after treatment (Tx) (methylprednisolone) (bottom); white dashed circles mark colocalization of the MMPI-PET signal and contrast enhancement in the MRI scan, which resolves by 35 days after treatment, correlating with a residual MMPI-PET signal. Left: Gd-T1-MRI. Middle: [^18^F]**15** scan 30 to 60 min after injection co-registered to the Gd-T1-MRI scan. Right: An overlay of Gd-T1-MRI and (PET-MRI). (**B**) Patient #2 represents an acute attack of RRMS. Left: Single co-registered [^18^F]**15** (30 to 60 min after injection) and Gd-T1-MRI slice at baseline (top) and 28 days after treatment (1 g of methylprednisolone, three times per day) (bottom). Right: Five consecutive PET (upper row) and corresponding MRI (bottom row) slices at baseline (top panel), 7 days after therapy (middle panel, PET only), and 28 days after therapy (bottom panel). Note the intense contrast enhancement in Gd-T1-MRI (maximum, white dashed circle #1) with spatially adjacent but not identical MP activity (black circle #1) at baseline. Reduced MMPI-PET signal at 7 days after therapy (black circle #2), and by 28 days after therapy, the MMPI-PET signal remains low (black circle #3), but the Gd-T1-MRI signal now colocalizes to the original MMP active area from the baseline scan. Reprinted with permission from [103] Copyright © 2016, American Association for the Advancement of Science.

They synthesized [^18^F]**15** using the method previously described by Wagner et al. [104] and used it for translational PET imaging in MS patients. A total of five patients with known or suspected relapsing-remitting MS (RRMS) presenting acute neurological symptoms participated in a [^18^F]**15**-PET study (Figure 9). All patients with the up-regulation of activated MMP-9 in cerebrospinal fluid samples exhibited a higher uptake of [^18^F]**15** into the brain (Figure 9). 

## 5. MMPIs Labelled Imaging Agents for Rheumatoid Arthritis (RA), and Osteo Arthritis (OA)

**Scheme 15:**
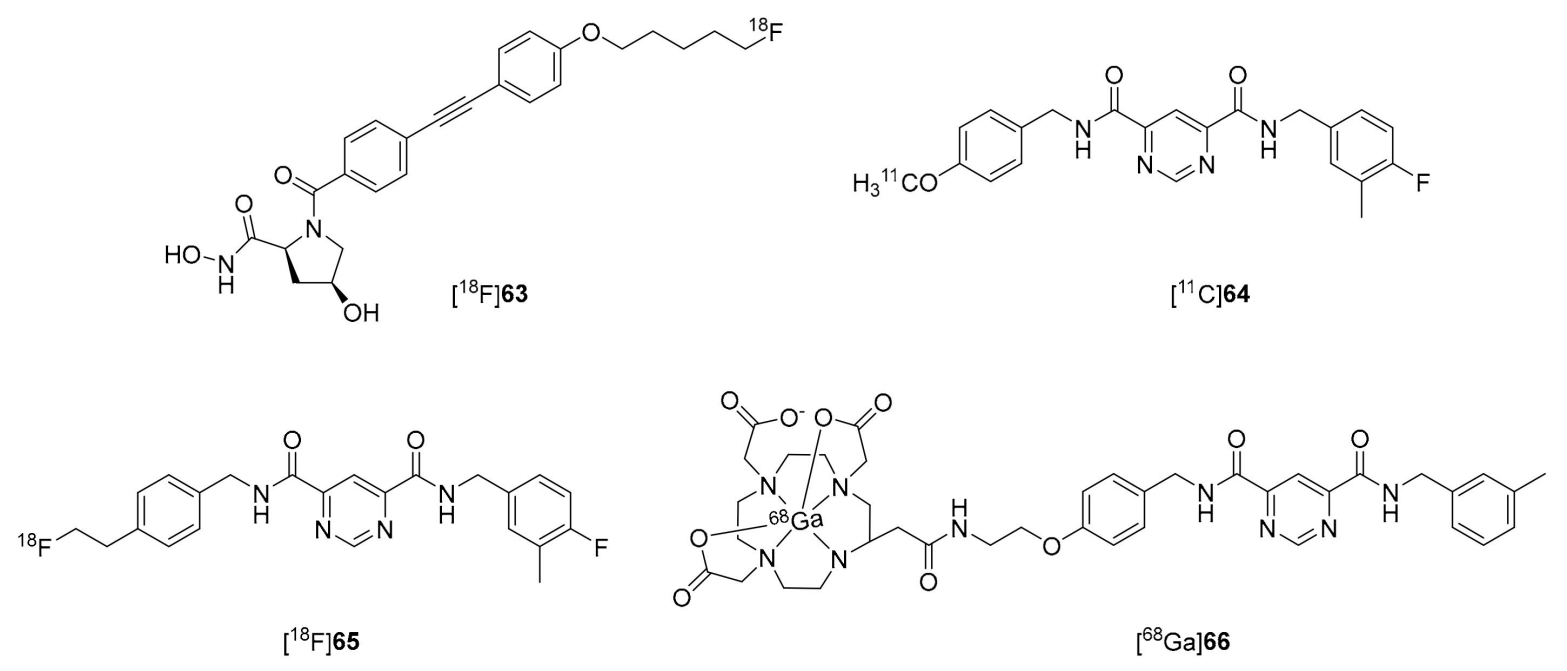
Chemical structure for compounds **63**–**66**.

MMP-13 is overexpressed in the cartilage of patients with OA. The unlabeled Compound **63** (Scheme 15) is a picomolar MMP-13 inhibitor (IC_50_ = 0.07 nM) with high selectivity towards other MMPs and ADAMs [105]. Interestingly, [^18^F]**63** exhibited excellent serum stability, and favorable in vivo biodistribution, which may be useful for PET imaging in RA and OA.

Selective and potent MMP-13 inhibitors **64**–**66** were synthesized by Hugenberg et al. and successful radiolabeling was performed with ^11^C, ^18^F, and ^68^Ga, respectively [106]. Although [^11^C]**64** and [^18^F]**65** showed moderate metabolic stability, the more hydrophilic [^68^Ga]**66** showed an enhanced blood retention time in biodistribution studies. Nearly no elimination of the [^68^Ga]**66** tracer-associated activity from the liver was detected, which lead the authors to assume that the tracer somehow accumulates in the liver, due to the metabolism. They concluded that radiolabeled selective MMP-13 inhibitors-based PET tracers could enable earlier and more specific diagnosis of several MMP-13 related diseases, such as OA and RA.

## 6. MMPIs Labelled Imaging Agents for Chronic Obstructive Pulmonary Disease (COPD) and Lung Inflammation

**Figure 10:**
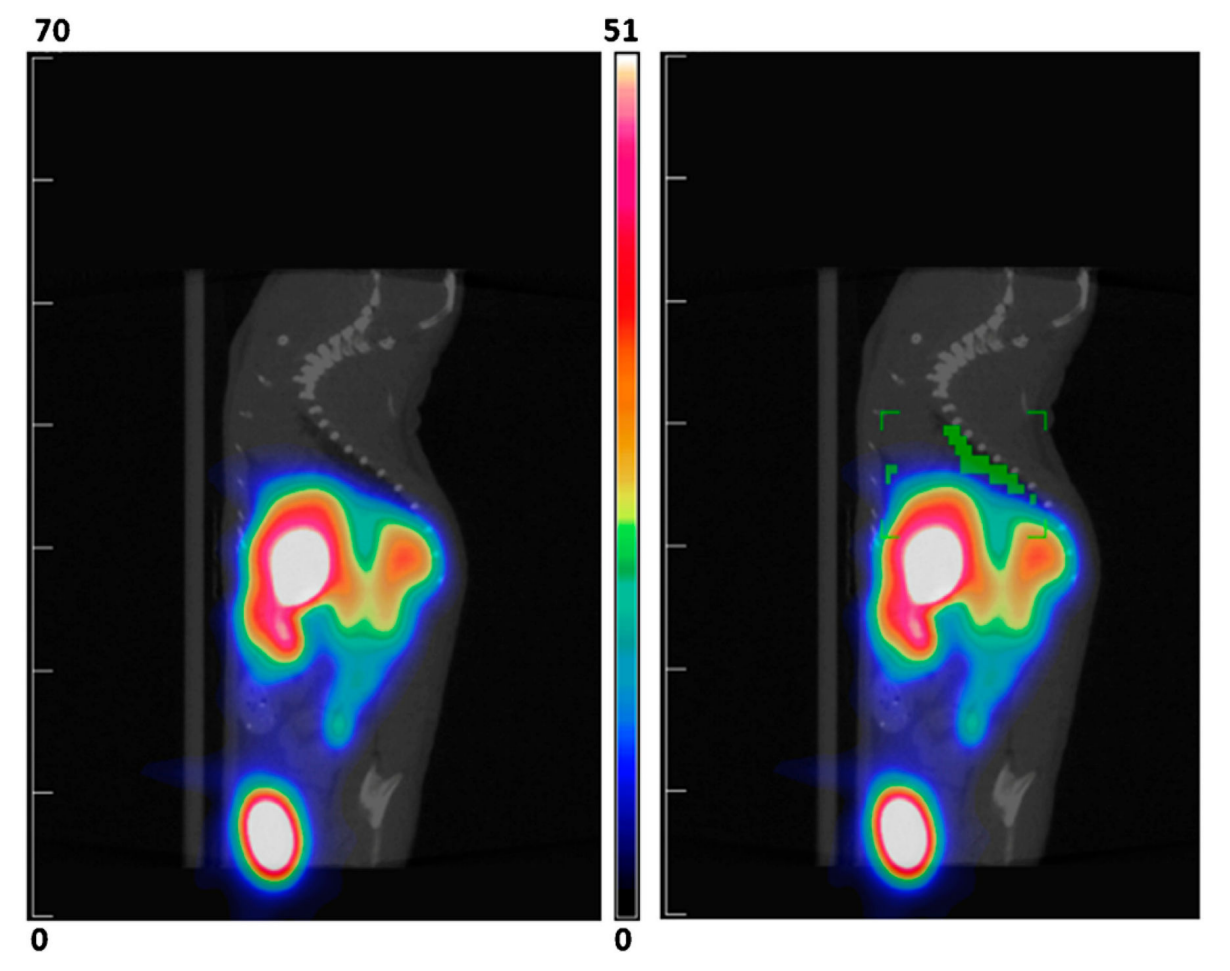
In vivo [^18^F]**45** microPET/CT images of a mouse exposed to cigarette smoke (CS) shown in a sagittal view. The right view indicates the ROIs (Regions Of Interest) drawn in the lung. The microPET images correspond to the sum of all the frames from 12 to 90 min p.i. of [^18^F]**45**. Reprinted with permission from [107] Copyright © World Molecular Imaging Society 2015.

The synthesis and application of [^18^F]**45** in cancer was described by Elsinga and colleagues as described before [92]. In 2015, they studied its utility in COPD [107]. Dynamic microPET studies in air or cigarette smoke (CS) exposed BALB/c mice showed a 2-fold increased the uptake in mice exposed to CS compared to air exposed mice (Figure 10). 

**Scheme 16:**
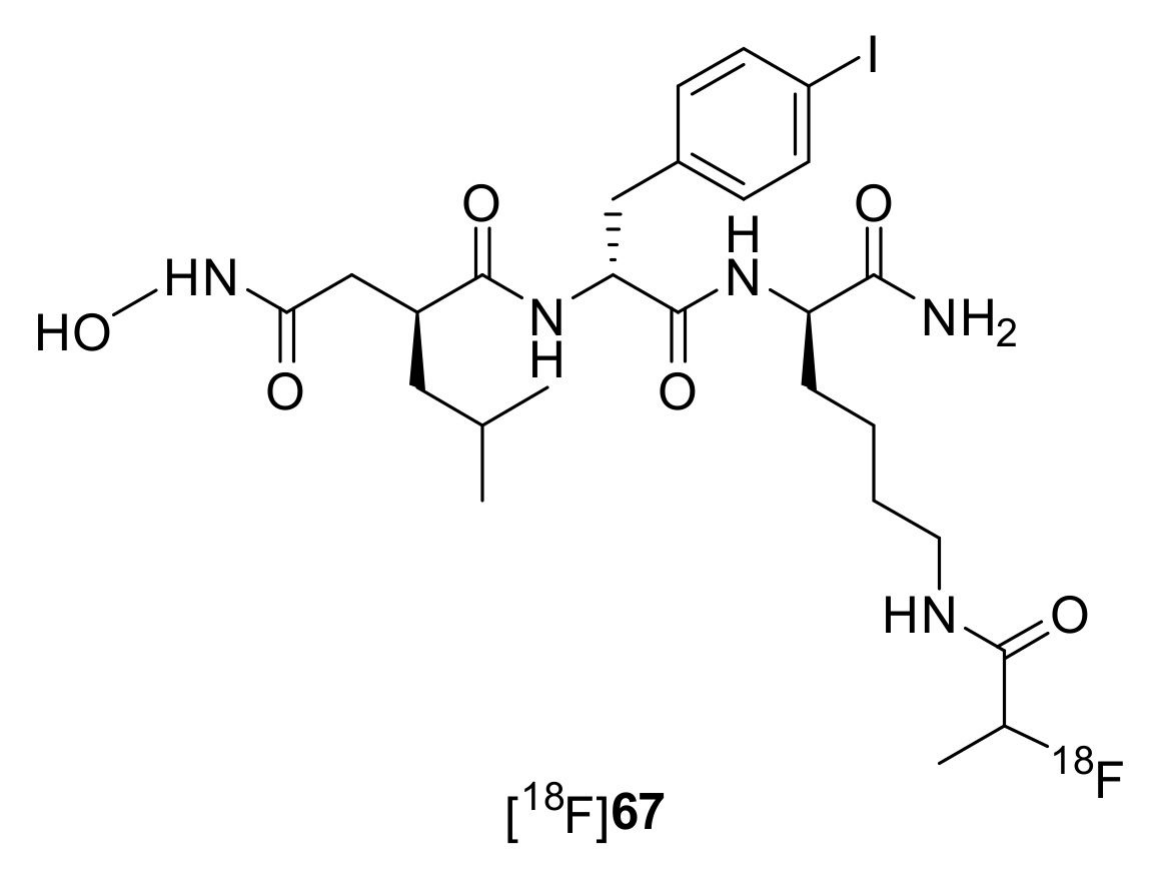
Chemical structure **67**.

**Figure 11:**
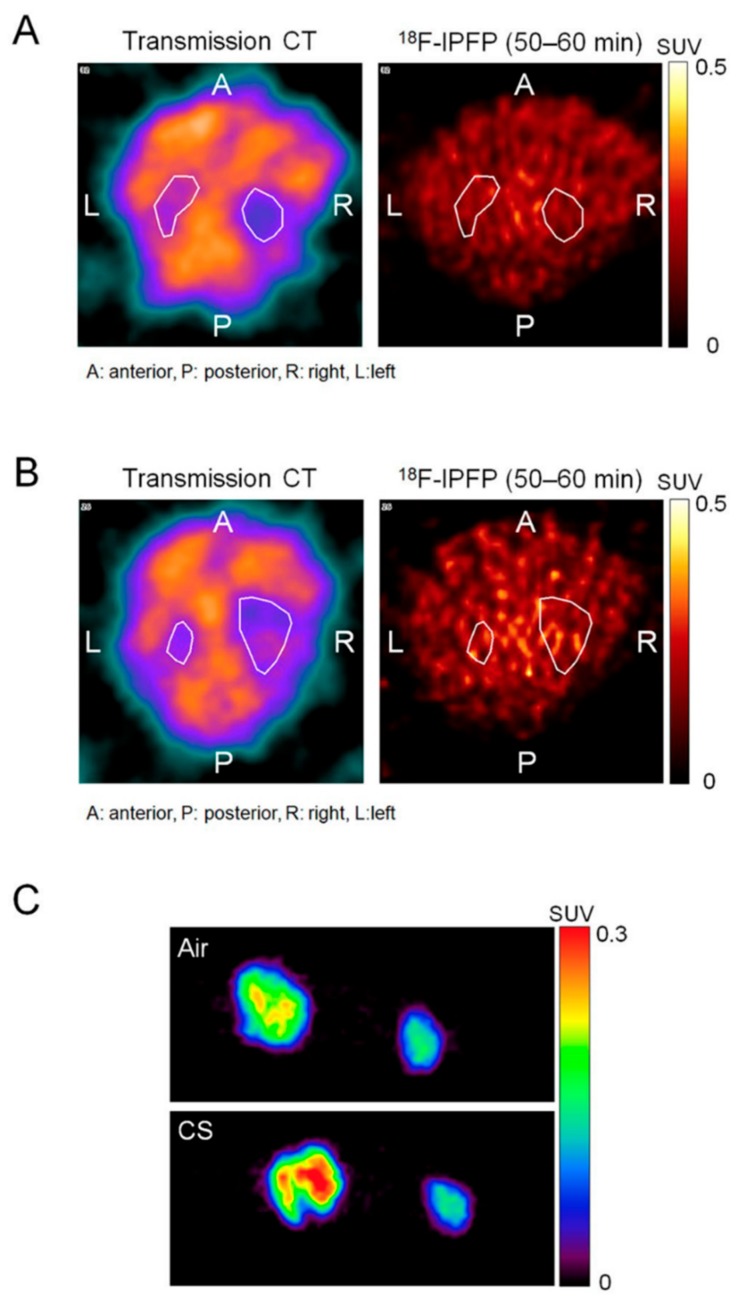
Transverse view of transmission CT and PET images at 50-60 min after injection of [^18^F]**67** IPFP in the (**A**) Air and (**B**) CS groups. (**C**) Maximum intensity projection images of removed lungs (left) and heart (right) from the Air (upper) and CS (lower) groups. Reprinted with permission from [108] copyright © 2018, Springer Nature.

Temma et al. developed [^18^F]**67** (Scheme 16) for MMPs-targeted PET [108]. Non-radiolabeled **67** exhibited IC_50_ in the nanomolar range (MMP-2 = 4.4, MMP-9 = 8.1, MMP-12 = 1.5, MMP-13 = 4.1 nM). The in vivo and ex vivo PET confirmed a 2-fold increased accumulation in the CS exposed lungs compared to normal mice (Figure 11).

A macrocyclic hydroxamate based radiotracer [^99m^Tc]**59** was mentioned before for its utility in early vascular imaging and AAA [109,110]. Further, in vivo and ex vivo studies of [^99m^Tc]**59** in murine models of COPD demonstrated its higher uptake in lung tissue. 

**Scheme 17:**
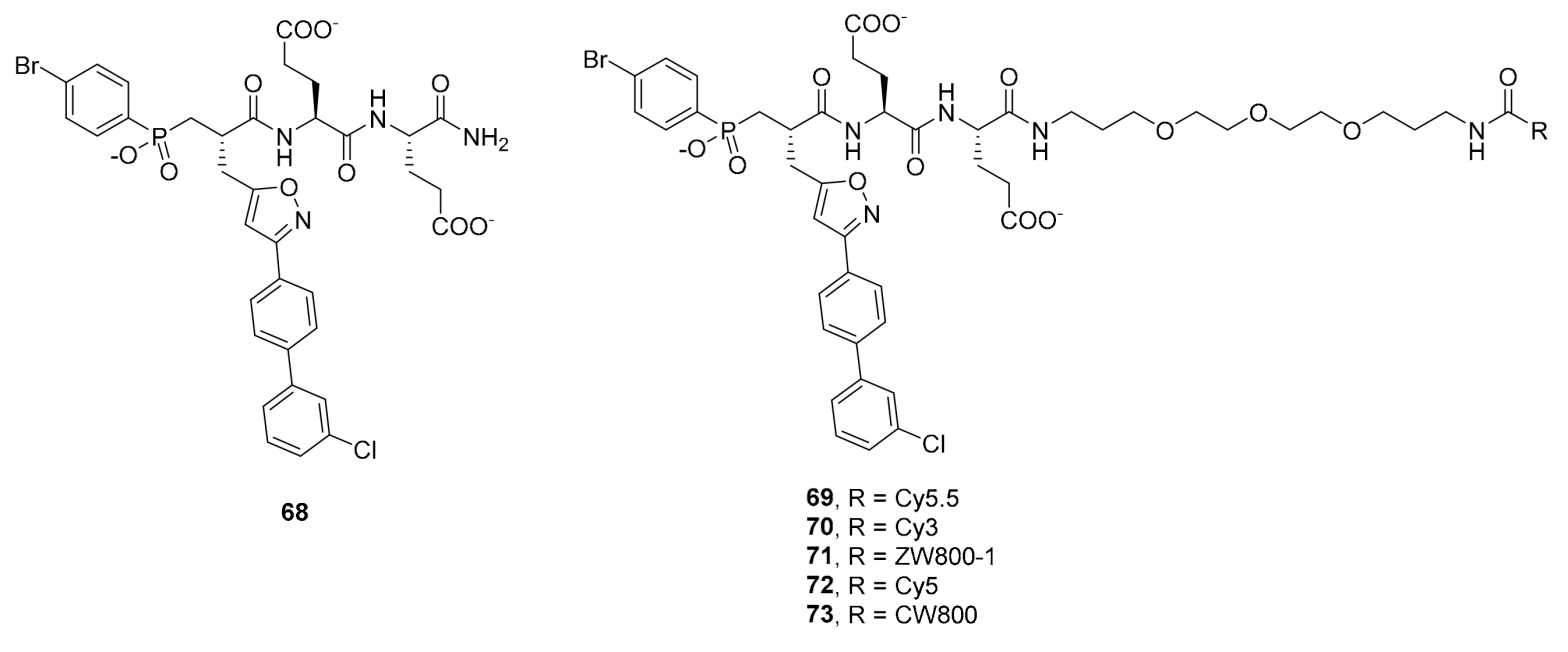
Chemical structure for compounds **68**–**73**.

Sadeghi et al. [111] developed fluorescent probes (**69**–**73**) (Scheme 17) to image active MMP-12 in inflammation and aneurysm. Among these probes, **70** (*K_i_* = 6.1 nM) and **71** (*K_i_* = 3.4 nM) presented the higher potency and selectivity towards MMP-12. Animals injected with **71** showed the highest signal in previously implanted sterile polyvinyl material (sponges) harvested for 60 min and a specific aneurysmal carotid artery when compared to a non-specific (scramble) control probe in WT mice. 

**Scheme 18:**
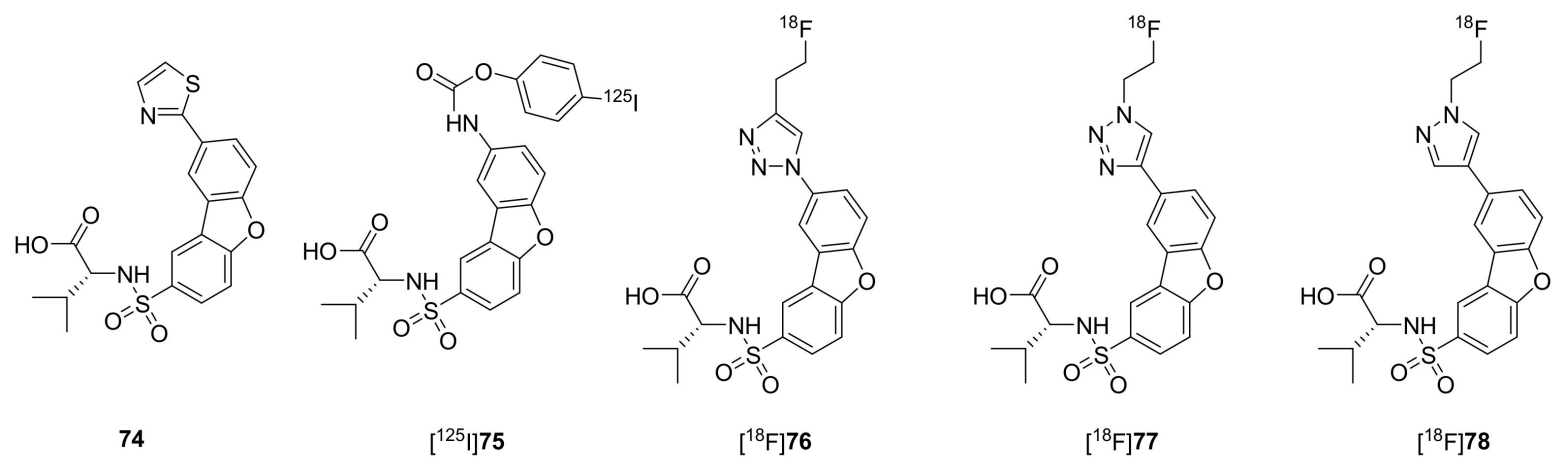
Chemical structure for compounds **74**–**78**.

Mukai and col. [112] described **74** (Scheme 18), a dibenzofuran sulfonamide-based MMPI. Interestingly, due to the specific interactions with the S1’ pocket, compound **74** exhibited more than a 150-fold activity for MMP-12 over MMP-8 and -13. Moreover, it prevented lung inflammation induced by recombinant human MMP-12 in C57BL/6 mice [113]. Inspired by these results, Mukai and colleagues designed and synthesized novel MMP-12-targeted dibenzofuran radiolabelled compounds with a variety of linker structures such as carbamate, amide, and sulfonamide. Among these, the best results were obtained for the carbamate linker present in compound [^125^I]**75** (IC_50_ = 8.5 nM).

Wagner and colleagues designed and synthesized a series of dibenzofuran unlabeled sulfonamides **76**–**78** [36]. These compounds revealed excellent inhibitory activities (IC_50_ values: 0.0004−0.19 nM) and high selectivity (≥25 fold) for MMP-12 and to date, they are the most potent MMP-12 inhibitors described in the literature.

Preliminary biodistribution and PET studies in C57BL/6 mice showed that the corresponding radiotracers [^18^F]**76**–**78** exhibited no specific uptake in non-excretion organs, indicating them as promising MMP-12 based PET imaging agents. 

## 7. Summary and Future Perspectives

In summary, in last two decades, several fluorescent-labelled or radio-labelled MMPIs have been developed as imaging agents for different diseases. Remarkably, pilot-clinical studies of [^18^F]**15** in MS patients described by Wagner et al. form an excellent basis for the feasibility of MMPI probes toward clinical trials [104]. A combined in vitro and in vivo evaluation revealed that molecular imaging probes based on MMPIs could have potential as validated imaging agents, but further research is necessary to reach clinical application. Although biodistribution studies of certain MMPI radiotracers in wild type mice have been described, further extensive SPECT/PET preclinical studies with proper controls in murine disease models (at least 5 mice per group), metabolite formation, stability studies, and toxicity studies are essential to confirm their clinical utility. 

The clinical translation of MMPs for PET or SPECT imaging as well as other prognostic and diagnostic biomarkers, requires time and is very costly. Therefore, it is crucial to select the candidates that have the highest possibilities of success to be translated into the clinic, based not only in the abovementioned preclinical models and pharmacokinetic studies, but also on economic perspectives. 

## 8. Conclusions

The overexpression of different MMPs has been associated with a number of relevant diseases such as cancer, atherosclerosis, stroke, arthritis, cardiovascular diseases, periodontal disease, respiratory tract disorders, glomerulonephritis, abdominal aortic aneurysm expansion, inflammatory bowel disease, neurodegeneration, chronic obstructive pulmonary disease, multiple sclerosis and liver fibrosis. In recent years, many MMPIs have been developed as novel therapeutics for these disorders, with some of them having entered clinical trials. More recently, numerous MMPIs labeled imaging agents have emerged as powerful tools for the early diagnosis and assessment of the efficacy of currently approved therapies. The structure-based design of highly selective MMPIs broadens the possibilities of succeeding in the development of clinically useful imaging tools. This knowledge, gathered by a wide number of research groups, would undoubtedly allow the validation of specific MMPs as relevant biomarkers in different diseases.

This review summarizes the main achievements in the field of MMPIs imaging probes and constitutes an important contribution to support further research in this area. Our research group has designed, in the last years, highly potent and selective inhibitors of MMP-2, while avoiding other MMPs, including the highly homologous gelatinase MMP-9 [114,115,116,117,118]. The structure based drug design on MMP-13 carried out by the authors has also provided very interesting and highly selective inhibitors [119]. All these compounds constitute an important starting point for the development of imaging probes. Based on this experience, the authors are currently outlining the design strategies to obtain ^18^F marked analogues for PET and fluorochrome derivatives for fluorescent detection. 

As highlighted in this review, a combination of medicinal chemistry and chemical biology tools has become essential for the detection of diseases in the early stages remarkably improving the treatments. MMPs have demonstrated to be relevant biomarkers for several of the most prevalent diseases. Although many contributions have been reported to date, a deeper knowledge of the involvement of these metalloenzymes will afford, in the near future, small molecule candidates as molecular imaging probes which can be useful in the clinical practice.

## Figures and Tables

**Table 1:** A comparison of various imaging modalities available for clinical imaging purposes.

	PET	SPECT	MRI	CT	US	OI
Spatial Resolution	1–2 mm	1–2 mm	25–100 μm	50–200 μm	50–500 μm	2–3 mm
Penetration Depth	limitless	limitless	limitless	limitless	mm to cm	cm
Time Resolution	sec–min	min	min–hr.	min	sec–min	sec–min
Principle	γ-rays	γ-rays	Radio waves	x-rays	High frequency Sound waves	Visible and Near infrared light
Sensitivity	high	high	poor	high	poor	poor
Probe Quantity	ng	ng	μg-mg	-	μg–mg	μg–mg
Probes	^18^F, ^11^C, ^68^Ga, ^64^Cu	^99^mTc, ^123^I, ^125^I, ^111^In, ^177^Lu	Gd, SPIO, Fe_3_O_4_, MnO	I_2_, Ba, Xe, Kr	microbubbles	Fluorescent dye and NIR dyes
Risk	Ionizing Radiation	Ionizing Radiation	No radiation	Ionizing Radiation	No radiation	No radiation

**Table 2:** The experimental details in the radiosynthesis of matrix metalloproteinases inhibitors (MMPIs) based imaging probes.

Probe	Probe Type	Total Synthesis Time	Radio Chemical Yield	Radio Chemical Purity	Activity at the End of the Synthesis	log P/log D	In Vitro Assay Results (Cold or Hot Compound)
**1** **2** **3**	^11^C ^18^F ^11^C	20–25 min	40–60%	-	-	-	-
**4**–**8**	^11^C	20–25 min	40–60%	92–98%	0.6–0.8 Ci/μmol	-	-
**9** **10**	^11^C ^11^C	-	20–30% 15–25%	>99% >95%	0.6–0.8 Ci/μmol 0.6–0.8 Ci/μmol	-	-
**11** **12** **13** **14**	^123^I ^125^I ^123^I ^125^I	-	6.1% 22.8% 41.7% 65.9%	>95% >95% >95% >95%	237.3 Ci/μmol 2.1 Ci/μmol 237.2 Ci/μmol 2.2 Ci/μmol	-	Unlabelled Inhibitor MMP-2 = 2.5 nM MMP-9 = 4.6 nM Unlabelled Inhibitor MMP-2 = 57.5 nM MMP-9 = 257 nM
**15** **16**	^18^F ^18^F	113 min 115 min	12.4% 45.6%	>97% >95.5%	32–72 GBq/μmol 42–97 GBq/μmol	clog P = 4.04 clog D = 4.03 clog P = 2.54 clog D = 2.53	MMP-2 = 4 nM MMP-8 = 2 nM MMP-9 = 50 nM MMP-13 = 11 nM MMP-2 = 4 nM MMP-8 = 2 nM MMP-9 = 50 nM MMP-13 = 11 nM
**17**	^18^F	160–180 min	12.1%	98.8%	39 GBq/μmol	-	MMP-2 = 8 nM MMP-8 = 0.9 nM MMP-9 = 0.5 nM MMP-13 = 0.9 nM
**18**	^18^F	95–11 min	31–46%	98%	3.5 GBq/μmol	**18**-(*R*) clog D = 2.23 **18**-(*S*) log D = 1.16	**18**-(R) MMP-2 = 10.4 nM MMP-9 = 0.5 nM **18**-(S) MMP-2 = 0.3 nM MMP-9 = 0.1 nM
**19**	^18^F	110–120 min	30%	98%	14–57 GBq/μmol	log D = 0.60 clog D = 1.53	MMP-2 = 0.13 nM MMP-8 = 0.02 nM MMP-9 = 0.03 nM MMP-13 = 0.006 nM
**20**	^18^F	97 min	47&	98%	9–46 GBq/μmol	log D = 2.25 clog D = 1.25	MMP-2 = 0.7 nM MMP-8 = 0.4 nM MMP-9 = 0.07 nM MMP-13 = 0.05 nM
**26**	^11^C	15–20 min	40–55%	>99%	0.6–0.8 Ci/μmol	-	-
**27** **28**	^123^I ^123^I	-	55–65% 65–75%	>98% >98%	> 50 Ci/μmol > 58 Ci/μmol	-	Unlabelled Inhibitor MMP-2 = 9.3 nM MMP-9 = 201 nM MMP-2 = 0.6 nM MMP-9 = 2.4 nM
**29** **30**	^123^I ^123^I	-	65–75% 55–65%	>98% >98%	> 50 Ci/μmol > 58 Ci/μmol	-	Unlabelled Inhibitor MMP-2 = 23 nM MMP-9 = 429 nM MMP-2 = 48 nM MMP-9 = 740 nM
**31**–**37**	^11^C	20–25 min	40–60%	>95%	0.6–0.8 Ci/μmol	-	Parent inhibitor MMP-1 = 1.5 μM MMP-2 = 0.003 μM MMP-3 = 0.008 μM MMP-7 = 7.2 μM MMP-9 = 2.2 μM MMP-13 = 0.006 μM
**38**	^11^C	20–25 min	35–55%	>99%	0.6–0.8 Ci/μmol	-	-
**39**	^11^C	40 min	50–60%		11–26 GBq/μmol	-	MMP-2 = 110 nM MMP-9 = 200 nM
**40** **41**	^18^F ^18^F	70 min 60 min	10 3%	98 100	5 GBq/μmol 12 GBq/μmol	clog P = 4.44 clog D = 1.46 clog P = 3.76 clog D = 0.67	MMP-1 = 18,500 nM MMP-2 = 16 nM MMP-3 = 7100 nM MMP-9 = 107 nM MMP-12 = 44 nM MMP-13 = 22 nM MMP-12 = 1600 nM MMP-1 = 6100 nM MMP-2 = 61 nM MMP-3 = 6800 nM MMP-9 = 1500 nM MMP-12 = 500 nM MMP-13 = 220 nM MMP-14 = 4600 nM
**42**	^125^I		49%	> 90%	0.146 GBq/μmol (2.176 Ci/μmol)	3.68	MMP-2 = 7 nM MMP-9 = 2 nM
**43**	^18^F	142 mi	11.3%	98	23 GBq/μmol	clog D = 2.88	MMP-2 = 23 nM MMP-8 = 138 nM MMP-9 = 7 nM MMP-13 = 207 nM
**44**	^18^F	140 min	43%	>97%	11–20 GBq/μmol	-	MMP-2 = 58 nM MMP-8 = 58 nM MMP-9 = 27 nM MMP-13 = 51 nM
**45**	^68^Ga	-	87 % Post-labelling 66 % Pre-labelling	97 % Post-labelling 76 % Pre-labelling	-	-	-
**46** **47**	^123^I ^124^I		28% 44%	95 93	0.2–6.3 GBq/μmol 0.4–14.0 GBq/μmol	clog P = 1.40 clog D = 0.92	MMP-2 = 29 nM MMP-8 = 1170 nM MMP-9 = 1.3 nM MMP-13 = 362 nM
**51**	^18^F	3 h	13–16%	95%	41–66 GBq/μmol	-	Precursor FB-ML5 MMP-2 = 12.5 nM MMP-9 = 31.5 nM MMP-12 = 138.0 nM
**53**	^123^I	-	41.7%	95%	16.2 GBq/μmol	-	MMP-2 = 298 nM MMP-9 = 153 nM
**67**	^18^F	150 min	1.3%	99%	1.5 TBq/μmol	-	MMP-2 = 4.4 nM MMP-9 = 8.1 nM MMP-12 = 1.5 nM MMP-13 = 4.1 nM
**52**	^18^F	119 min	37	98	3–33 GBq/μmol	log D 1.65	Precursor Ki, MMP-2 = 103 nM MMP-12 = 221 nM MMP-9 = 2376 nM MMP-13 = 530 nM
**60**	^18^F	-	10–20	95	>90 GBq/μmol (>2.5 Ci/μmol)	-	MMP-2 = 1.8 nM MMP-9 = 7.2 nM
**62**	^123^I	-	38–63%	98	-	-	MMP-1 = 910 nM MMP-2 = 0.87 nM MMP-9 = 1.95 nM
**63**	^18^F	105 min	46.3	99	0.7–33.2 GBq/μmol	log D = 2.50 clog D = 1.18	MMP-2 = 5 nM MMP-8 = 21 nM MMP-9 = 108 nM MMP-13 = 0.07 nM
**64** **65** **66**	^11^C	29 min 75 min 74 min	36–50% 20–23% 73%	98% 98% 99	122–330 GBq/μmol 18–35 GBq/μmol 18–20 GBq/μmol	log D = 3.35 log D = 2.77 log D = −1.12	MMP-2 = >100 nM MMP-8 = >100 nM MMP-9 = >100 nM MMP-13 = 56 nM MMP-14 = >100 nM MMP-2 = >100 nM MMP-8 = >100 nM MMP-9 = >100 nM MMP-13 = 26 nM MMP-14 = >100 nM MMP-2 = >100 nM MMP-8 = >100 nM MMP-9 = >100 nM MMP-13 = 1.8 nM MMP-14 = >100 nM
**75**	^125^I	-	91%	98%	5.1 GBq/μmol	-	MMP-12 = 8.5 nM
**76** **77** **78**		126 min 140 min 161 min	37% 43 22	99 99 99	3–59 GBq/μmol 7–57 GBq/μmol 3–68 GBq/μmol	log D = 0.48 log D = 0.00 log D = 0.63	MMP-2 = 81 nM MMP-8 = 19 nM MMP-9 = 2230 nM MMP-12 = 0.19 nM MMP-13 = 288 nM MMP-2 = 35 nM MMP-8 = 2 nM MMP-9 = >10,000 nM MMP-12 = 0.001 nM MMP-13 = 16 nM MMP-2 = 0.84 nM MMP-8 = 0.18 nM MMP-9 = 15.1 nM MMP-12 = 0.0004 nM MMP-13 = 3.2 nM
